# Universal Path-Following of Wheeled Mobile Robots: A Closed-Form Bounded Velocity Solution †

**DOI:** 10.3390/s21227642

**Published:** 2021-11-17

**Authors:** Reza Oftadeh, Reza Ghabcheloo, Jouni Mattila

**Affiliations:** 1 Department of Computer Science and Engineering, Texas A&M University, College Station, TX 77840, USA; 2 Department of Automation Technology and Mechanical Engineering, Tampere University, 33720 Tampere, Finland; reza.ghabcheloo@tuni.fi (R.G.); jouni.mattila@tuni.fi (J.M.)

**Keywords:** wheeled mobile robots, path-following, nonholonomic constraints

## Abstract

This paper presents a nonlinear, universal, path-following controller for Wheeled Mobile Robots (WMRs). This approach, unlike previous algorithms, solves the path-following problem for all common categories of holonomic and nonholonomic WMRs, such as omnidirectional, unicycle, car-like, and all steerable wheels. This generality is the consequence of a two-stage solution that tackles separately the platform path-following and wheels’ kinematic constraints. In the first stage, for a mobile platform divested of the wheels’ constraints, we develop a general paradigm of a path-following controller that plans asymptotic paths from the WMR to the desired path and, accordingly, we derive a realization of the presented paradigm. The second stage accounts for the kinematic constraints imposed by the wheels. In this stage, we demonstrate that the designed controller simplifies the otherwise impenetrable wheels’ kinematic and nonholonomic constraints into explicit proportional functions between the velocity of the platform and that of the wheels. This result enables us to derive a closed-form trajectory generation scheme for the asymptotic path that constantly keeps the wheels’ steering and driving velocities within their corresponding, pre-specified bounds. Extensive experimental results on several types of WMRs, along with simulation results for the other types, are provided to demonstrate the performance and the efficacy of the method.

## 1. Introduction

Wheeled Mobile Robots (WMRs) form a significant subset of Unmanned Ground Vehicles (UGVs). The continuing demand for more advanced and autonomous UGVs entails more reliable and higher-performance motion controllers for WMRs. The multitude of motion controllers proposed for mobile robots, especially those with nonholonomic constraints, may be roughly categorized into three groups [1]: point stabilization [2], trajectory tracking [3], and path-following [4]. Typically, the path-following approach is used under a decoupled control architectures [5], where a path-planner provides the desired geometric path. Then, a path-following module, while considering the temporal and other intrinsic constraints of the system, maneuvers the robot toward the planned path and steers it so that it indefinitely follows the path.

The path-following algorithms are classified into several branches, including, but not limited to, Optimal Control approaches [6,7], Feedback Linearizion methods [8], Line of Sight guidance laws [9], Pure Pursuit techniques [10], and Vector Fields methods [11] (see [12] for a thorough review). The majority of these algorithms incorporate a concept that goes by many names, including “Virtual Target Point”, “Carrot”, and “Rabbit”. In this concept, a virtual point is selected and moved along the desired path, while a tracking controller, also called a guidance controller, makes the robot follow and converge to that point. The differences among and between those branches mainly occur in the design of the guidance controller, the method for selecting the virtual point, and the its motion strategy along the path.

### 1.1. Related Work

This work belongs to a category of path-followers that parametrize (using the path’s natural parameter) a virtual position for the mobile robot on the desired path. A nonlinear guidance controller is employed for the robot to track the virtual point based on an error space projected on the path through the path’s Serret–Frenet frame.

Early notable works in this field were conducted by [13] for the car-like WMRs, by [14] for the unicycle types, and by [15] for both unicycles and WMRs with two steering wheels. However, the  projection scheme of this approach, which selects the path’s closest point to the robot as the virtual point, would result in singularities that, in turn, would impose stringent initial conditions on the system and on the drivable path curvatures. This drawback was overcome by having the path’s natural parameter as an auxiliary state and, therefore, deriving a control law for its progression rate along the path [16]. There have been various extensions of the original problem, such as covering uncertainties [17], actuator saturations [18], obstacle avoidance [19] and an extension to aerial [20], marine  [21] and articulated frame [22] vehicles. Comprehensive experimental results of some of these algorithms have been provided by [23]. However, the majority of recent studies on the path-following of WMRs consider only a special type of WMR: usually the unicycle type, with some exceptions, such as [24]. Nevertheless, there is no unified solution for the path-following of WMRs.

Aside from the general difficulties in designing a universal path-follower for WMRs, this paper tackles several challenges in the design of motion controllers that are *specific* to certain types of WMRs. Most notable is the presence of singularities, both inherently [25] and in the representation of the configuration space [26] of WMRs with active steering wheels. While several types of WMR possess steering wheels, those singularities are a major issue for WMRs that utilize two or more actively steered standard wheels. Due to their kinematic configuration, such WMRs are called nonholonomic omnidirectional robots [27,28] or pseudo-omnidirectional robots [29,30]. They have recently gained a significant level of popularity and are now being used in a wide range of practical fields, including service robotics (PR2 [31],Care-O-bot [32],Rollin’ Justin [33]), space robotics (Mars-Exo-Rover [30]), agricultural applications [34,35], among others [36].

The common way of formulating the configuration space of such WMRs is with the notion of Instantaneous Center of Rotation (ICR) [37]. As the ICR moves closer to a wheel axis, the driving velocity of that wheel decreases, while the curvature of the wheel’s footprint, and, hence, its steering velocity, unboundedly increases. When the ICR coincides with the wheel, its steering angle becomes undefined and singular. To circumvent such singular configurations, many proposed solutions rely on numerical methods to plan singularity-free ICR trajectories in velocity space [25]. Others treat the neighborhood of the singularities as obstacles and solve a navigation problem [29,38]. However, in all of those methods, considerable portions of the configuration space are avoided, thus reducing the maneuverability of the platform. Furthermore, when the robot is required to follow a desired path and heading profile, ICR position has already been determined and, therefore, none of those approaches are suitable for path-following problems.

### 1.2. Contributions and Organization

The contributions of this paper are as follows:

- This study solves the path-following problem for all WMRs categories in which their wheels roll without skidding. To the best knowledge of the authors, this is the first study that coherently solves the path-following problem with this level of generality.
- Unlike other path-followers, in this design, the control signals and the resultant vector field of closed-loop equations of motion are *linearly proportional* to the base speed (In this paper, speed exclusively refers to the magnitude of velocity vectors.). In fact, the controller acts as a feedback path-planner that minimizes the Lyapunov function of errors as its corresponding cost function.
- The kinematic constraints of all types of wheels are rigorously derived in their most general form. We derive and prove sufficient conditions for a path-following controller that simplify the kinematic and nonholonomic constraints into explicit relations between the speed of the base and that of the wheels.
- Based on this framework, we present a *closed-form* solution for the speed of the WMR’s base so that all the wheels’ steering and driving speeds remain within their respective bounds. We show that the solution is time-optimal, because it provides a bang-bang velocity profile in which, at each time step, at least one of the wheels runs at its maximum speed.
- This solution allows WMRs with active steering wheels to get close to, and even pass, their singular configurations by regulating the speed of the WMR and the steering velocities of the wheels. Hence, this method expands the allowable configuration space of such robots and allows them to exploit their whole maneuverability.

This paper is the culmination of several earlier studies presented by the authors in [39,40,41]. Compared to [41], this paper has several novelties. Section 3 is new, in which the controller in [41] is generalized into a generic parametrized form and we demonstrate that the controller serves as an example of such generic form. Moreover, the results of [40] are coherently included to address the singularities of WMRs with steering wheels. The majority of the equations, especially the kinematic constraints, are derived in a more general form and are presented in compact matrix format that is further consistent with the formulation of WMR constraints in the literature. A new set of comprehensive experimental and simulation results in a more complex scenario is presented, with further explanations and remarks.

This paper is organized as follows. In Section 2, we formally define the WMR that is the focus of this paper and define the corresponding path-following problem. Next, in Section 3, we present the path-following solution in a parametrized generic form. In Section 4, the variables in the parametrized model are meticulously derived and categorized for different types of wheels and WMRs. We explain how the solution solves the problem of singularities for WMRs with steering wheel in Section 4.3 and, finally, Section 5 covers the implementation results and provides extensive experimental data for three types of WMRs, and simulation results for the other two.

## 2. Problem Description

WMRs are classified based on the seminal work of [42] into five kinematically feasible categories. An ordered pair $\left(\delta_{m},\delta_{s}\right)$ is assigned to each category the degree of mobility and degree of steerability of the WMR, respectively. The number of wheels, their types, and their arrangements determine $\delta_{m}$, and $\delta_{s}$ and, therefore, the WMRs’ category. $\delta_{m}$ represents the dimension of the tangent space of the configuration space, while $\delta_{s}$ corresponds to the ability to change (steer) the basis of the tangent space by means of steering wheels. The degree of maneuverability defined as $\delta_{M}=\delta_{m}+\delta_{s}$ then, analogous to degrees of freedom for mechanism, determines the total mobility of the WMR.

### 2.1. WMR Architecture and Definitions

**Definition** **1** (WMR). *The WMR considered in this paper is equipped with n wheels, wced the* base*. It belongs to and possesses the minimum actuated wheels of one of those five kinematically feasible categories of WMRs, and the actuators provide velocity and position control. Each wheel is of one of the following types:* fixed wheels, standard steerable wheels, off-centered steerable wheels (Caster wheels), Swedish wheels. *Furthermore, the following assumptions hold. The WMR traverses on a flat and horizontal plane. The base and the wheels are rigid, the tires are non-deformable, their contact surface with the ground can be approximated with a point, and there is no mechanical constraint for the steering of the steerable wheels; hence, they are free-turn. However, we will discuss the case with limited steering angle separately in Section 5.3.*

**Figure 1:**
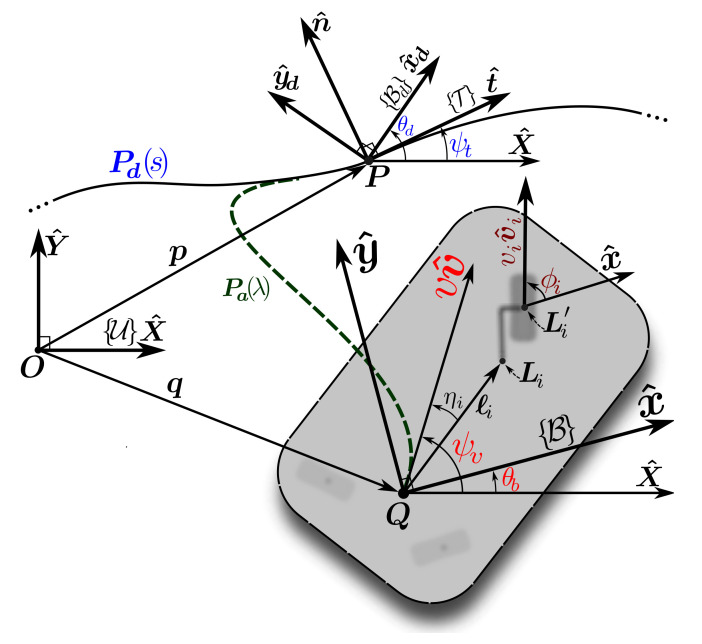
The desired path and the required coordinate frames.

Figure 1, depicts a schematic view of a WMR and the desired path. Additionally, Table 1 lists the definitions of the corresponding parameters and variables. We define the base current posture X, as 

(1) $$X={\left[\begin{array}{cc} q^{T} & \theta_{b} \end{array}\right]}^{T},$$

where q is the position vector of point Q (the origin of body frame B) and $\theta_{b}$ is the heading angle. The base linear velocity at Q is $v\hat{v}$ with *v* being the base speed and $\hat{v}\left(\psi_{v}\right)$ being the direction of the velocity as a function of the linear velocity angle $\psi_{v}$. Moreover, based on Definition 1, for a general WMR, several types of wheels may be connected to the base. As shown in Figure 1, the connection point of the *i*th wheel is $L_{i}$ and the velocity of the wheel is $v_{i}{\hat{v}}_{i}$, in which $v_{i}$ is the driving speed and ${\hat{v}}_{i}\left(\phi_{i}\right)$ is the direction of wheels heading, which, in turn, is a function of wheel’s steering angle $\phi_{i}$.

The desired path $P_{d}$ is a 2D and bounded-curvature regular curve on the horizontal plane. This is defined by the vector-valued function $P_{d}\left(s\right):\left[0,L_{d}\right]\to R^{2}$, where *s* and $L_{d}$ are the natural parametrization (arc length) and the length of $P_{d}$, respectively. The desired heading function $\theta_{d}\left(s\right):\left[0,L_{d}\right]\to R$ of class $C^{3}$ determines the base’s desired heading $\theta_{d}$. Similar to Equation (1), the WMR desired posture, $X_{d}$, is defined as

(2) $$X_{d}={\left[\begin{array}{cc} P_{d}^{T} & \theta_{d} \end{array}\right]}^{T}.$$

Furthermore, the path $P_{a}\left(\lambda \right)$ is an asymptotic path to $P_{d}$. It is defined by $P_{a}\left(\lambda \right):\left[0,L_{a}\right]\to R^{2}$, where λ and $L_{a}$ are the natural parametrization (arc length) and length of the path, respectively, with its tangent angle denoted by $\psi_{a}$. Correspondingly, the function $\theta_{a}\left(\lambda \right):\left[0,L_{d}\right]\to R$ is an asymptotic angle from the base current heading $\theta_{b}$, to the desired heading $\theta_{d}$. We will later show that $P_{a}\left(\lambda \right)$ and $\theta_{a}\left(\lambda \right)$ are solutions to autonomous differential equations with the current pose of the base as the initial conditions.

### 2.2. Problem Formulation

As shown in Figure 1, the virtual target point on $P_{d}$ is denoted as P. It is determined by *s*, which is set as an *auxiliary* state with $\dot{s}$ being its corresponding control signal. Along with *s*, we define error state variables as

(3a) $$\begin{array}{cc} S & =\left(x_{e},y_{e},\theta_{e},\psi_{e}\right) \end{array}$$

(3b) $$\begin{array}{cc} S^{*} & =\left(x_{e},y_{e},\theta_{e}\right), \end{array}$$

where,

(4a) $$\begin{array}{cc} \left[\begin{array}{c} \;x_{e}\;\;\\ \;y_{e}\;\; \end{array}\right]\; & ={{}^{U}\;R}_{T}^{-1}\left(q-p\right) \end{array}$$

(4b) $$\begin{array}{cc} \theta_{e} & =\theta_{d}-\theta_{b}, \end{array}$$

(4c) $$\begin{array}{cc} \psi_{e} & =\psi_{d}\left(s,S^{*}\right)-\psi_{v}, \end{array}$$

and ${{}^{U}\;R}_{T}$, equivalent to $R(\psi_{t})$, is the rotation matrix from frame T to frame U. In the above, the position error signals, $x_{e}$ and $y_{e}$, are measured along $\hat{t}$ and $\hat{n}$, respectively, while $\theta_{e}$ independently represents the heading error. The signal $\psi_{d}$, as a function of $S^{*}$, is the desired heading that is determined by the controller and basically generates a suitable approach angle to $P_{d}\left(s\right)$.

The time derivation of Equations (4a) to (4c) yields to the open-loop equations of motion

(5a) $$\begin{array}{cc} {\dot{x}}_{e} & =\dot{s}\left(\kappa_{d}\left(s\right)y_{e}-1\right)+v\cos\left(\psi_{t}-\psi_{v}\right) \end{array}$$

(5b) $$\begin{array}{cc} {\dot{y}}_{e} & =-\dot{s}\kappa_{d}\left(s\right)x_{e}-v\sin\left(\psi_{t}-\psi_{v}\right) \end{array}$$

(5c) $$\begin{array}{cc} {\dot{\theta }}_{e} & =\frac{d\theta_{d}}{ds}\dot{s}-\omega_{b} \end{array}$$

(5d) $$\begin{array}{cc} {\dot{\psi }}_{e} & ={\dot{\psi }}_{d}-\omega_{v}, \end{array}$$

in which the angular velocity of the frame B, is $\omega_{b}≜{\dot{\theta }}_{b}$ and, similarly, $\omega_{v}≜{\dot{\psi }}_{v}$. We assemble all of the above formulations into the following definition for the WMR’s base.

**Definition** **2** (Base Path-Following). Base path-following error dynamics is a system with the full states (s,S) or the reduced states $\left(s,S^{*}\right)$, and with the control inputs C, which are defined as

(6) $$C=\left(\dot{s},\omega_{b},\omega_{v}\right).$$

*The dynamics of this system is given by Equations (5a) to (5d), in which the base speed v is seen as an exogenous input.*

Some further notes are given here for the reduced states, $S^{*}$. For the majority of WMRs, except a special case, it is possible to incorporate a rather simpler scheme by dismissing $\psi_{e}$ in (4c) as an error state ($\psi_{e}=0\;\forall t$), therefore controlling the base velocity direction $\hat{v}\left(\psi_{v}\right)$ directly. In the reduced states $S^{*}$, $\psi_{v}$ acts as a control signal by directly setting $\psi_{v}=\psi_{d}\left(s,S^{*}\right)$. In Section 4.2, we will detail the types of WMRs and conditions under which the choice of $S^{*}$ is not possible.

In what follows, we formally define the problem that is the focal point of this paper.

**Problem** **1** (WMR Bounded Velocity Path-Following). *Given the desired path $P_{d}\left(s\right)$ and heading profile $\theta_{d}\left(s\right)$ derive feedback control laws for the wheels’ driving $v_{i}$ and steering inputs $\phi_{i}$ (or their rates ${\dot{\phi }}_{i}$) such that:*

 *(I)*  *Path-Following: The velocity frame $B_{v}$ converges and follows the tangent frame T; that is, error signals $x_{e}$ and $y_{e}$, remain bounded and converge to zero (See Equation (4a)).*
 *(II)*  *Heading Control: The body frame B converges and follows $B_{d}$; that is, the heading error signal $\theta_{e}$, remains bounded and converges to zero (See (4b)).*
 *(III)*  *Bounded Velocity: $v_{i}$ and ${\dot{\phi }}_{i}$ should not exceed their corresponding predefined limits.*

We solve the above problem in two stages. In the first stage, we place our focus solely on the base path-following defined by Definition 2, its stability and features. In the second stage, we focus on the kinematic constraints between the wheels and the base, the details of which are given in Section 3.2. We utilize these constraints to solve sub-problems (I) and (II) of Problem 1 by mapping the intermediary control inputs C, to the wheels’ variables. Furthermore, those mappings are used to solve the bounded velocity problem (sub-problem (III)) by selecting an appropriate *v* that bounds the driving and steering velocities.

At first glance, this approach is solely applicable to holonomic omnidirectional WMRs ($\delta_{m}=3$). However, this treatment is a rather general and we will show that, for each category of WMRs, a proper subset of the above intermediary control sets along with a pertinent choice of body origin Q results in a feasible solution that abides by the kinematic limitations of that category.

## 3. WMR Path-Following: The Generic Form

In the following, Section 3.1 focuses on a generic form of C for the base path-following that has some unique features. Then, in Section 3.3, those features are utilized to present a parametrized version of the WMRs’ kinematic constraints, which, in turn, are incorporated into a closed-form solution for Problem 1.

### 3.1. Base Path-Following

**Proposition** **1.** *For the base path-following system defined by Definition 2, assume that some exists feedback control laws exist for C that render the origin of the error space asymptotically stable and they are in the generic form of*

(7a) $$\begin{array}{cc} \dot{s} & =s^{\prime }\left(s,S\right)v \end{array}$$

(7b) $$\begin{array}{cc} \omega_{b} & =\kappa_{b}\left(s,S\right)v \end{array}$$

(7c) $$\begin{array}{cc} \omega_{v} & =\kappa_{v}\left(s,S\right)v \end{array}$$

(7d) $$\begin{array}{cc} \psi_{d} & =\psi_{d}\left(s,S^{*}\right), \end{array}$$

*in which $s^{\prime }$, $\kappa_{b}$, and $\kappa_{v}$ are functions of *only* s and error states, S. Then, at any given time t≥0, the closed-loop equations of motion result in a set of differential equations for an asymptotic path $P_{a}\left(\lambda \right)$ and a heading $\theta_{a}\left(\lambda \right)$, with the initial conditions being q(t) and $\theta_{b}\left(t\right)$, respectively. In other words, X(t), the base posture at the time t, is*

(8) $$X\left(t\right)={\left[\begin{array}{cc} P_{a}^{T}\left(\lambda =0\right) & \theta_{a}\left(\lambda =0\right) \end{array}\right]}^{T},$$

*and as λ increases, ${\left[\begin{array}{cc} P_{a}^{T}\left(\lambda >0\right) & \theta_{a}\left(\lambda >0\right) \end{array}\right]}^{T}$ asymptotically converges toward the desired posture $X_{d}$.*

**Proof.**  To prove the above proposition, notice that $\left||\dot{q}|\right|=v$ and a geometric variable λ exist, where $\dot{\lambda }=v$. Consequently, Equation (7a) becomes $\dot{s}=s^{\prime }\dot{\lambda }$. Based on the chain rule, we have $v=\frac{ds}{d\lambda }\dot{\lambda }$ and, hence, $s^{\prime }=\frac{ds}{d\lambda }$. Using the same analogy for Equations (7b) and (7c) and substituting them into the open-loop error states (Equations (5a) to (5d)) results in

(9a) $$\begin{array}{cc} \frac{ds(\lambda )}{d\lambda } & =s^{\prime }\left(s,S\right) \end{array}$$

(9b) $$\begin{array}{cc} \frac{dx_{e}\left(\lambda \right)}{d\lambda } & =x_{e}^{\prime }\left(s,S\right)=s^{\prime }\left(\kappa_{d}\left(s\right)y_{e}-1\right)+\cos\left(\psi_{t}-\psi_{v}\right) \end{array}$$

(9c) $$\begin{array}{cc} \frac{dy_{e}\left(\lambda \right)}{d\lambda } & =y_{e}^{\prime }\left(s,S\right)=-s^{\prime }\kappa_{d}\left(s\right)x_{e}-\sin\left(\psi_{t}-\psi_{v}\right) \end{array}$$

(9d) $$\begin{array}{cc} \frac{d\theta_{e}\left(\lambda \right)}{d\lambda } & =\theta_{e}^{\prime }\left(s,S\right)=\frac{d\theta_{d}}{ds}s^{\prime }-\kappa_{b}\left(s,S\right) \end{array}$$

(9e) $$\begin{array}{cc} \frac{d\psi_{e}\left(\lambda \right)}{d\lambda } & =\psi_{e}^{\prime }\left(s,S\right)=\psi_{d}^{\prime }-\kappa_{v}\left(s,S\right), \end{array}$$

where,

(10) $$\psi_{d}^{\prime }=\frac{d\psi_{d}}{d\lambda }=\frac{\partial \psi_{d}}{\partial x_{e}}x_{e}^{\prime }+\frac{\partial \psi_{d}}{\partial y_{e}}y_{e}^{\prime }+\frac{\partial \psi_{d}}{\partial \theta_{e}}\theta_{e}^{\prime }\;.$$

The above equations provide a set of differential equations for (s,S) based on the independent variable λ. The algebraic Equations (4a) to (4c) can be used to track the geometric evolution of q, $\theta_{b}$, and $\psi_{v}$ as functions of λ, which are $P_{a}\left(\lambda \right)$, $\theta_{a}\left(\lambda \right)$, and $\psi_{a}\left(\lambda \right)$, respectively, and are governed by

(11a) $$\begin{array}{cc} P_{a}\left(\lambda \right) & =P_{d}\left(s\left(\lambda \right)\right)+{{}^{U}\;R}_{T}\left(s\left(\lambda \right)\right)\;\left[\begin{array}{c} \;x_{e}\left(\lambda \right)\;\;\\ \;y_{e}\left(\lambda \right)\;\; \end{array}\right] \end{array}$$

(11b) $$\begin{array}{cc} \theta_{a}\left(\lambda \right) & =\theta_{d}\left(s\left(\lambda \right)\right)-\theta_{e}\left(\lambda \right) \end{array}$$

(11c) $$\begin{array}{cc} \psi_{a}\left(\lambda \right) & =\psi_{d}\left(s\left(\lambda \right),S^{*}\left(\lambda \right)\right)-\psi_{e}\left(\lambda \right). \end{array}$$

Therefore, the differential Equations (9a) to (9e) together with the algebraic Equations (11a) to (11c) result in a set of expressions for the asymptotic path $P_{a}\left(\lambda \right)$ and heading $\theta_{a}\left(\lambda \right)$. Notice that λ does not explicitly appear in any of the above equations; therefore, (s(t),S(t)), the base path-following states at time *t*, can be associated with initial conditions (λ=0) of the above differential equations.    □

The merit of Proposition 1 is that a majority of path-following controllers in the literature (e.g., [16,19]) cannot be written in the generic form of Equations (7a) to (7c). Therefore, while, in those path-followers, *v* is an exogenous input and does not have a direct role in the stability (as long as $v\geq v_{m}>0$), the asymptotic path $P_{a}$ cannot be determined independently of v(t) and it is only after the assignment of the speed profile that one can derive the asymptotic trajectory $P_{a}\left(\lambda \left(t\right)\right)$. However, Proposition 1 enables us to determine $P_{a}\left(\lambda \right)$ without specifying the future velocity commands by directly integrating closed-loop equations. In other words, a path-following controller in the form of Equations (7a) to (7d) acts as a feedback path-planner that has a certain error function as its cost function and plans asymptotic paths from the base current posture X, toward the desired posture $X_{d}$. We provide an example for such a controller in Section 4.1.

In this paper, there is no need to solve the closed-loop differential equations. The differentials of (s,S), obtained in the form of the above proposition, will be used for WMRs’ kinematic constraints in the next proposition. For that purpose, the higher time differentiations of control signals and error states may also be written as differentials based on λ, which are listed below for future reference.

(12a) $$\begin{array}{cc} \ddot{s} & =s^{\prime \prime }\left(s,S,S^{\prime }\right)v^{2}+s^{\prime }\left(s,S\right)\dot{v} \end{array}$$

(12b) $$\begin{array}{cc} {\dot{\omega }}_{b} & =\kappa_{b}^{\prime }\left(s,S,S^{\prime }\right)v^{2}+\kappa_{b}\left(s,S\right)\dot{v} \end{array}$$

(12c) $$\begin{array}{cc} {\dot{\omega }}_{v} & =\kappa_{v}^{\prime }\left(s,S,S^{\prime }\right)v^{2}+\kappa_{v}\left(s,S\right)\dot{v}, \end{array}$$

where, $s^{\prime \prime }=\frac{ds^{\prime }}{d\lambda }=\frac{d^{2}s}{d\lambda^{2}}\;$, $\kappa_{b}^{\prime }=\frac{d\kappa_{b}}{d\lambda }\;$, $\kappa_{v}^{\prime }=\frac{d\kappa_{v}}{d\lambda }\;$, and 

(13) $$S^{\prime }=\left(x_{e}^{\prime },y_{e}^{\prime },\theta_{e}^{\prime },\psi_{e}^{\prime }\right).$$

Finally, for the closed-loop system, we may transform ${}^{B}\;\dot{X}$ and ${}^{B}\;\ddot{X}$, the velocity and acceleration of the base posture (Equation (1)) expressed in the body frame B, into 

(14a) $$\begin{array}{cc} {}^{B}\;\dot{X} & ={}_{\;}^{B}{\;X}^{\prime }v,\;{}^{B}\;\dot{X^{\prime }}={}_{\;}^{B}{\;X}^{\prime \prime }v, \end{array}$$

(14b) $$\begin{array}{cc} {}^{B}\;\ddot{X} & ={}_{\;}^{B}{\;X}^{\prime \prime }v^{2}+{}_{\;}^{B}{\;X}^{\prime }\dot{v}, \end{array}$$

(14c) $$\begin{array}{cc} {}_{\;}^{B}{\;X}^{\prime } & ={\left[\begin{array}{ccc} \cos\psi_{vb} & \sin\psi_{vb} & \kappa_{b}) \end{array}\right]}^{T}, \end{array}$$

(14d) $$\begin{array}{cc} {}_{\;}^{B}{\;X}^{\prime \prime } & ={\left[\begin{array}{ccc} -\kappa_{vb}\sin\psi_{vb} & \kappa_{vb}\cos\psi_{vb} & \kappa_{b}^{\prime } \end{array}\right]}^{T}, \end{array}$$

in which, $\psi_{vb}-2pt≜\psi_{v}-\theta_{b}$, and $\kappa_{vb}-2pt≜\kappa_{v}-\kappa_{b}$. The above transformation facilitates the treatment of kinematic constraints in Section 3.2.

### 3.2. Wheels’ Kinematic Constraints

**Figure 2:**
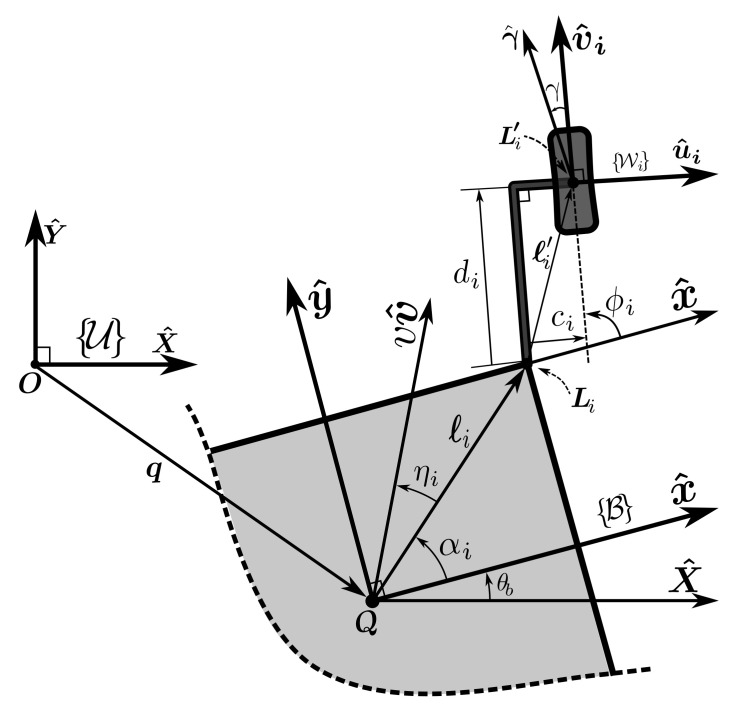
A generalized wheel and its corresponding parameters.

Figure 2 depicts a schematic view of an abstract Generalized Wheel (GW) as the *i*th wheel of the WMR and its corresponding parameters. The GW represents both Swedish wheels and normal wheels. In this sense, $r_{sr}$ and γ define the radius and the direction of the small rollers’ axis, respectively; hence, for a functional Swedish wheel: $\gamma \neq \frac{\pi }{2}$ and $r_{sr}\neq 0$. For a normal, non-Swedish wheel, we simply set $\gamma =\frac{\pi }{2}$ and $r_{sr}=0$. The wheel is mounted on an L-shaped rod, parametrized by off-center values: $\left(d_{i},c_{i}\right)$, at the point $L_{i}^{\prime }$, with ${{}_{\;}^{B}\ell^{\prime }}_{i}=c_{i}\;{}_{\;}^{B}{\hat{u}}_{i}+d_{i}\;{}_{\;}^{B}{\hat{v}}_{i}$. The rod is connected to the base at the attachment point $L_{i}$ by a revolute joint. As shown in the figure, $\phi_{i}$ represents the steering angle of the wheel, and $v_{i}{\hat{v}}_{i}$ represents its *driving velocity vector*, generated by the wheel’s actuator. We define the absolute steering angle $\Phi_{i}$ and the projection matrix $J_{i}\left({\hat{x}}_{i}\right)$ as

(15a) $$\begin{array}{cc} \Phi_{i} & =\theta_{b}+\phi_{i}, \end{array}$$

(15b) $$\begin{array}{cc} J_{i}\left({\hat{x}}_{i}\right) & =\left[\begin{array}{cc} {\hat{x}}_{i}^{T} & {\hat{x}}_{i}.\left(\hat{z}\times {}^{B}\ell_{i}\right) \end{array}\right], \end{array}$$

in which ${\hat{x}}_{i}$ is an arbitrary unit vector.

Moreover, for fixed and Swedish types, the steering direction ${}_{\;}^{B}{\hat{v}}_{i}\left(\phi_{i}\right)$, is a mechanical design variable, and is measured for steerable types, except when it is set as the control signal—a case that will be further explored. Table 2, for each type of wheel, lists the required values for the GW variables and parameters. To derive the kinematic relations for the GW, we may differentiately form the vector relation $L_{i}^{\prime }=q+\ell_{i}+d_{i}{\hat{v}}_{i}+c_{i}{\hat{u}}_{i}$ and express it in the body frame to arrive at a velocity constraint between the wheel and the base, which is

(16) $$\begin{array}{cc} v\;\;{}^{B}\hat{v}+\omega_{b}\left(\hat{z}\times {}^{B}\ell_{i}\right)= & \left(v_{i}-c_{i}{\dot{\Phi }}_{i}\right)\;\;{}^{B}\hat{v_{i}}+d_{i}{\dot{\Phi }}_{i}\;\;{}^{B}\hat{u_{i}}\\ \; & -r_{sr}{\dot{\phi }}_{sr}\left(\hat{z}\times {}_{\;}^{B}\hat{\gamma }\right), \end{array}$$

in which ${\dot{\phi }}_{sr}$ is the angular velocity of the GW’s small rollers. The above equation can be manipulated into scalar equations

(17) $$v_{i}\;{\hat{a}}_{i}.\;{}_{\;}^{B}{\hat{v}}_{i}=J_{i}\left({\hat{a}}_{i}\right)\;\;{}^{B}\;\dot{X}+{\dot{\Phi }}_{i}{\hat{a}}_{i}.\left(\hat{z}\times {{}_{\;}^{B}\ell^{\prime }}_{i}\right)$$

where,

(18) $${\hat{a}}_{i}=\left\{\begin{array}{cc} {}_{\;}^{B}\hat{\gamma }\left(\gamma \right)\; & \mathrm{Swedish}\;\mathrm{wheel}(\gamma \neq \frac{\pi }{2},r_{sr}\neq 0)\\ {}_{\;}^{B}{\hat{v}}_{i}\left(\phi_{i}\right)\; & \mathrm{Other}\;\mathrm{types}(\gamma =\frac{\pi }{2},r_{sr}=0) \end{array}\right..$$

Equation (17) accompanied by the proper choice of ${\hat{a}}_{i}$ (Equation (18)) determines the driving velocity $v_{i}$. By definition, for fixed and Swedish wheels ${\dot{\phi }}_{i}=0$. For the steerable wheels, when $d_{i}\neq 0$, ${\dot{\phi }}_{i}$ is determined by setting ${\hat{a}}_{i}={}_{\;}^{B}{\hat{u}}_{i}$ in Equation (17), which eliminates the left-hand side of the equation. When ${}_{\;}^{B}{\hat{u}}_{i}.\left(\hat{z}\times {{}_{\;}^{B}\ell^{\prime }}_{i}\right)$ is zero (equivalent to $d_{i}=0$), setting ${\hat{a}}_{i}={}_{\;}^{B}{\hat{u}}_{i}$ and $d_{i}=0$ in Equation (17) and differentiating from this yields:

(19) $${\dot{\Phi }}_{i}J_{i}\left({}_{\;}^{B}{\hat{v}}_{i}\right)\;{}^{B}\;\dot{X}+J_{i}\left({}_{\;}^{B}{\hat{u}}_{i}\right)\;{}^{B}\;\ddot{X}=\omega_{b}^{2}\;\;{}_{\;}^{B}{\hat{u}}_{i}.{{}_{\;}^{B}\ell }_{i}.$$

Evidently, the above kinematic constraints cannot be analytically solved for a base speed that results in specified driving and steering velocities. However, as  in the following proposition, we show that the incorporation of Proposition 1 into the kinematic constraints yields a set of direct relationships between the base speed and wheel velocities.

**Proposition** **2.** *Consider a WMR defined in Definition 1. If the WMRs intermediary control signals C conform to the generic form, as outlined in Proposition 1, then the driving and steering velocities of the ith wheel (1≤i≤n) are in the form of*

(20a) $$\begin{array}{cc} v_{i} & =v_{i}^{\prime }\left(s,S,S^{\prime },C^{\prime }\right)v \end{array}$$

(20b) $$\begin{array}{cc} {\dot{\phi }}_{i} & =\phi_{i}^{\prime }\left(s,S,S^{\prime },C^{\prime }\right)v, \end{array}$$

*in which, $v_{i}^{\prime }=\frac{dv_{i}}{d\lambda }$, $\phi_{i}^{\prime }=\frac{d\phi_{i}}{d\lambda }\;$, and *

(21) $$C^{\prime }=\left(s^{\prime },\kappa_{b},\kappa_{v}\right).$$

**Proof.**  We prove this proposition by constructing $v_{i}^{\prime }$ and $\phi_{i}^{\prime }$. This is carried out by substituting the results of Proposition 1 into the kinematic constraints and performing some algebraic manipulations that can be simplified to the form of Equations (20a) and (20b). Based on Equations (7b) and (15a), we have ${\dot{\Phi }}_{i}=\omega_{b}+{\dot{\phi }}_{i}$ and $\omega_{b}=\kappa_{b}v$. Therefore, the proof for Equation (20b) is equivalent to finding an expression for ${\dot{\Phi }}_{i}=\Phi_{i}^{\prime }v$ and setting $\phi_{i}^{\prime }=\Phi_{i}^{\prime }-\kappa_{b}$. Clearly, Equation (20b) automatically holds for zero steering wheels (fixed and Swedish wheels) with $\Phi_{i}^{\prime }=\kappa_{b}$ and $\phi_{i}^{\prime }=0$. For the other types, setting ${\hat{a}}_{i}={}_{\;}^{B}{\hat{u}}_{i}$ in Equation (17), and substituting ${}^{B}\;\dot{X}$ by ${}_{\;}^{B}{\;X}^{\prime }v$ from Equation (14c), and ${}^{B}\;\ddot{X}$ from Equation (14b) in Equation (19), this can be rewritten in the form of ${\dot{\Phi }}_{i}=\Phi_{i}^{\prime }v$ with

(22) $$\Phi_{i}^{\prime }\;\;=\;\;\left\{\begin{array}{cc} d_{i}^{-1}J_{i}\left({}_{\;}^{B}{\hat{u}}_{i}\right){}_{\;}^{B}{\;X}^{\prime } & d_{i}\neq 0\\ {\left(J_{i}\left({}_{\;}^{B}{\hat{v}}_{i}\right){}_{\;}^{B}{\;X}^{\prime }\right)}^{{}^{-1}}\;\left(\kappa_{b}^{2}\;{}_{\;}^{B}{\hat{u}}_{i}.{{}_{\;}^{B}\ell }_{i}\;-\;J_{i}\left({}_{\;}^{B}{\hat{u}}_{i}\right){}_{\;}^{B}{\;X}^{\prime \prime }\right) & d_{i}=0 \end{array}\right.\;,$$

which proves the second relation, Equation (20b). Notice that, for $d_{i}=0$, the choice of control signals eliminates the acceleration terms from Equation (19) and simplifies it into the form of ${\dot{\Phi }}_{i}=\Phi_{i}^{\prime }v$. In this case, the only caveat is that $J_{i}\left({}_{\;}^{B}{\hat{v}}_{i}\right){}_{\;}^{B}{\;X}^{\prime }$ in the above equation may become very small, or even zero. This situation corresponds to the singularity configuration of wheels with $d_{i}=0$ that are called centered steering wheels. This situation and its treatment is fully explained in Section 4.3. Finally, following the same paradigm, Equation (17) can be manipulated into the form of Equation (20a) with

(23) $$v_{i}^{\prime }={\left({\hat{a}}_{i}.\;{}_{\;}^{B}{\hat{v}}_{i}\right)}^{-1}\;\left(J_{i}\left({\hat{a}}_{i}\right)\;\;{}_{\;}^{B}{\;X}^{\prime }+\Phi_{i}^{\prime }{\hat{a}}_{i}.\left(\hat{z}\times {{}_{\;}^{B}\ell^{\prime }}_{i}\right)\right),$$

in which ${\hat{a}}_{i}$ is determined by Equation (18).    □

**Remark** **1.** For a WMR with centered steerable wheels ($d_{i}=0$), under the reduced state model $S^{*}$, the wheels’ steering angles $\phi_{i}$ can be set as control signals and are derived as follows. Based on Equation (16), the steering direction is ${}_{\;}^{B}{\hat{v}}_{i}\left(\phi_{i}\right)$ is $v\;\;{}^{B}\hat{v}+\omega_{b}\left(\hat{z}\times {}^{B}\ell_{i}\right)$. Therefore, for the closed-loop system, $\omega_{b}$ can be replaced with $\kappa_{b}v$ and the steering direction becomes

(24) $${}_{\;}^{B}{\hat{v}}_{i}=\frac{{}^{B}\hat{v}+\kappa_{b}\left(\hat{z}\times {}^{B}\ell_{i}\right)}{\sqrt{1+\kappa_{b}^{2}l_{i}^{2}+2l_{i}k_{b}\sin\eta_{i}\;}}.$$

*The major benefit of the above formulation is that it determines the proper steering direction of the wheels, even when the robot is stopped and the base speed is zero.*

### 3.3. WMR Path-Following

**Figure 3:**
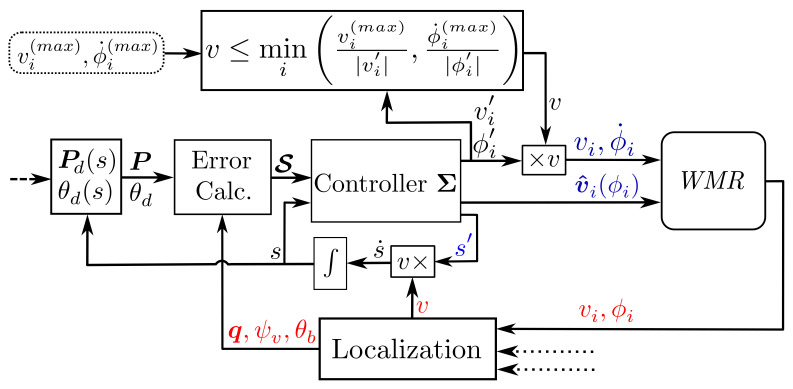
Schematic block diagram of the whole system.

The previous proposition most importantly shows that, for the closed-loop path-following of all WMR types, the kinematic constraints between the base and the wheels can be reduced to proportional functions of the base speed with proportions that are only functions of *s* and instant error states S. Consequently, a suitable solution to Subproblems (I) and (II) of Problem 1 is the selection of an arbitrary *v*, and a C that conforms to conditions in Proposition 1, and then using Equations (20a) and (20b) to evaluate the wheels’ driving and steering velocities. Furthermore, to solve the Subproblem (III), instead of having an arbitrary profile for *v*, Proposition 2 can be used to find instant limits for *v* that bound the wheels’ velocities. Such a solution exhaustively solves Problem 1 and is formally stated in the following Algorithm 1, with Figure 3 schematically depicting the corresponding block diagram.
**Algorithm 1:** WMR Bounded Velocity Path-Following.Assume that the WMR possess $n_{d}$ driving actuators and $n_{s}$ steering actuators ($n_{d}+n_{s}\leq 2n$). The maximum driving velocity of the *i*th driving actuator is denoted as $v_{i}^{\left(\max\right)}$, and the maximum steering velocity of the *i*th steering actuator is denoted as ${\dot{\phi }}_{i}^{\left(\max\right)}$. At each time step, the control signals of wheels’ actuators is evaluated by**Desired Inputs:** Evaluate the internal state *s*, by integrating the internal feedback $\dot{s}$, and obtain the virtual target values P and $\theta_{d}$ by using $P_{d}\left(s\right)$ and $\theta_{d}\left(s\right)$.**Error Calculation:** Evaluate the error states S by using the localization feedback (see Equations (4a) to (4c)).**Controller Σ:** Evaluate $S^{\prime }$ Equation (13), and $C^{\prime }$ Equation (21) and, by using the values of those signals, obtain ${\hat{v}}_{i}$, $v_{i}^{\prime }$, and $\phi_{i}^{\prime }$ of Equations (20a) and (20b) for all the wheels.**Bounded Velocity:** Based on Equations (20a) and (20b), there are $n_{d}+n_{s}$ candidates for *v*, namely, $v^{\left(i\right)}$, which are
(25)$$\begin{array}{cccccc} v^{\left(i\right)} & =\frac{v_{i}^{\left(\max\right)}}{|v_{i}^{\prime }|} & \; & \mathrm{and}\; & v^{\left(n_{d}+i\right)} & =\frac{{\dot{\phi }}_{i}^{\left(\max\right)}}{|\phi_{i}^{\prime }|} \end{array}$$Then, the maximum allowable base speed, denoted as $v^{\left(\max\right)}$, is
(26)$$v^{\left(\max\right)}=\min_{i}v^{\left(i\right)},\;i\in \left\{1,2,..,n_{d}+n_{s}\right\}.$$**Wheels’ Control Inputs:** Select a $v\leq v^{\left(\max\right)}$, and use $v_{i}=v_{i}^{\prime }v$ and $\dot{\phi_{i}}=\phi_{i}^{\prime }v$ to evaluate the actuators’ velocity commands (Equations (20a) and (20b)).

Note that Equations (20a) and (20b), which are used in the forth step of the above algorithm to derive the velocity candidates, are strictly monotonic with respect to $v_{i}$, and ${\dot{\phi }}_{i}$ and so is their inverse with respect to *v*. Hence, applying the minimum of those *m* velocity candidates results in driving and steering velocities less than or equal to the given velocity bounds. Alternatively, at each instant, if $v=v^{\left(\max\right)}$ is selected, then at least one of the actuators is being driven at its maximum velocity, which renders the solution a bang-bang control [43] for the velocity *v* and, therefore, for a given desired path, heading, and control gains, the solution is time-optimal.

## 4. WMR Path-Following: Detailed Illustration

In this section, we provide the pertinent expressions for the parametrized controller presented in the previous section. In Section 4.1, we present an example controller for the base that complies with the conditions of Proposition 1, and thereby customize Algorithm 1 for WMRs based on their degree of maneuverability, $\delta_{M}$.

### 4.1. Base Path-Following: The Controller

First, we define $\psi_{d}\left(s,S^{*}\right)$, the desired input for $\psi_{v}$ as

(27) $$\psi_{d}\left(s,S^{*}\right)=\psi_{t}-\sigma \left(y_{e}\right),$$

in which, $\sigma (y_{e})$ is a function that generates a suitable approach angle from the base to $P_{d}\left(s\right)$ and has the following features: σ(0)=0 and $y_{e}\sigma \left(y_{e}\right)>0\;\;\forall y_{e}\neq 0$. One candidate for $\sigma (y_{e})$ is

(28) $$\sigma \left(y_{e}\right)≜\sin^{-1}\frac{k_{2}y_{e}}{|y_{e}|+\epsilon },$$

where $0<k_{2}\leq 1$ and ϵ>0.

Based on the above definition, it can be construed that, for large normal errors $y_{e}$, $\sigma (y_{e})$ goes toward π/2 and, consequently, the base turns toward the virtual point P to decrease the error. As $y_{e}$ decreases, $\sigma (y_{e})$ moves toward zero and the base velocity turns toward the path tangent at P; therefore, the robot motion becomes more aligned with the path. Evidently, larger values for $k_{2}$ result in sharper turns for the robot to reach the path.

**Proposition** **3.** *The feedback control laws for signals C that are given by*

(29) $$\begin{array}{cccccc} \dot{s} & =s^{\prime }\left(s,S\right)v & \omega_{b} & =\theta_{b}^{\prime }\left(s,S\right)v & \omega_{v} & =\psi_{v}^{\prime }\left(s,S\right)v, \end{array}$$

*where,*

(30a) $$\begin{array}{cc} s^{\prime }\left(s,S\right) & =k_{1}x_{e}+\cos\left(\psi_{t}-\psi_{v}\right) \end{array}$$

(30b) $$\begin{array}{cc} \theta_{b}^{\prime }\left(s,S\right) & =k_{3}\theta_{e}+\frac{d\theta_{d}}{ds}s^{\prime } \end{array}$$

(30c) $$\begin{array}{cc} \psi_{v}^{\prime }\left(s,S\right) & =\psi_{d}^{\prime } \end{array}$$

(30d) $$\begin{array}{cc} \psi_{d}^{\prime } & =\kappa_{d}s^{\prime }-y_{e}^{\prime }\frac{d\sigma (y_{e})}{dy_{e}}. \end{array}$$

and $k_{1},k_{3}>0$, lead the reduced error states, $S^{*}$, to asymptotically converge to zero. Moreover, replacing Equation (30c) with

(31a) $$\begin{array}{cc} \psi_{v}^{\prime }\left(s,S\right) & =\kappa_{d}s^{\prime }-\frac{d\sigma (y_{e})}{dy_{e}}y_{e}^{\prime }-\kappa_{e}^{2}y_{e}\Delta +k_{4}\psi_{e} \end{array}$$

(31b) $$\begin{array}{cc} \Delta & =\left\{\begin{array}{cc} \frac{\sin(\psi_{t}-\psi_{v})-\sin\sigma (y_{e})}{\psi_{e}} & \;\psi_{e}\neq 0\\ \cos\sigma (y_{e}) & \;\psi_{e}=0 \end{array}\right., \end{array}$$

*in which $\kappa_{e}\neq 0\;\mathrm{and}\;k_{4}>0$ result in error states S asymptotically converging to zero. Consequently, the origin of the error space is stable and semi-globally exponentially stable by setting $v\left(t\right)\;\geq \;v_{m}\;>\;0\;\;\forall t$.*

**Proof.**  Here, we use standard quadratic form of error signals as the Lyapunov function but with modified control laws to make curvatures independent of speed, *v*. First, we provide a proof for the case where error states are $S^{*}$, and then we extend it for the full state S. Consider the following Lyapunov function:

(32) $$V_{1}=\frac{1}{2}x_{e}^{2}+\frac{1}{2}y_{e}^{2}+\frac{1}{2}\theta_{e}^{2},$$

which is positive, definite and radially unbounded. The time differentiation of $V_{1}$, along with the solution of Equations (5a) to (5c), results in:

(33) $${\dot{V}}_{1}=-\left(k_{1}x_{e}^{2}+k_{2}\frac{y_{e}^{2}}{|y_{e}|+\epsilon }+k_{3}\theta_{e}^{2}\right)\;v\left(t\right)\;,$$

which is negative; thus, the origin is stable. For a given $d_{1}>0$, if $v\left(t\right)\;\geq \;v_{m}\;>\;0$ and, initially, $|y_{e}\left(t_{0}\right)|<d_{1}$, it is easy to show that ${\dot{V}}_{1}<-\lambda V_{1}$. Thus, the origin is semi-globally exponentially stable.

To complete the proof, consider the following Lyapunov function:

(34) $$V_{2}=V_{1}+\frac{1}{2\kappa_{e}^{2}}\psi_{e}^{2}.$$

The time differentiation of $V_{2}$ along the solution of Equations (5a) to (5d) results in:

(35) $${\dot{V}}_{2}={\dot{V}}_{1}-\frac{k_{4}}{\kappa_{e}^{2}}\psi_{e}^{2}\;\;v\left(t\right)\;,$$

which, again, is negative; therefore, the origin of the error state is stable. □

The above control laws clearly follow the generic format of Proposition 1. As mentioned in the above proposition, with the given control laws, the origin of S is semi-globally exponentially stable for non-zero base speeds. The practical implication of this feature is that the WMR may stop (v=0) for some time during the path-following operation, during which the states remain bounded and the path-following is naturally resumed once the WMR starts to move and the speed is not zero anymore. Note that there are several other functions for $\sigma (y_{e})$ in the literature. However, while all of them result in negative $\dot{V}$, the one presented here is the one that results in a quadratic form for $y_{e}$ in $\dot{V}$, and hence provides exponential stability.

### 4.2. Customization of the Path-Following Algorithm

WMRs are classified based into five different categories on the ordered pair $\delta =(\delta_{m},\delta_{s})$. Three of these categories possess the degree of maneuverability $\delta_{M}=\delta_{m}+\delta_{s}=3$, and, for the other two, $\delta_{M}=2$. In the following, we customize Algorithm 1 based on the WMR’s degree of maneuverability and explain the accompanying details. The results of this section are listed in Table 3.

#### 4.2.1. WMRs with $\delta_{M}=3$

These types of WMRs are omnidirectional in nature, which means that they have independent heading and linear movements. However, the holonomic type with δ=(3,0) provides full mobility and, hence, instantaneous velocity in any direction. The other two categories (δ=(2,1)andδ=(1,2)) are steerable and nonholonomic. They are capable of providing movement in any arbitrary direction, but only after they have steered their wheels to the corresponding configuration. For the problem at hand, the difference between holonomic and nonholonomic types only occurs at the beginning of the path, in which the holonomic type may start path-following instantly, but the nonholonomic types have to rearrange their steerable wheels. Other than this, on a smooth path and heading profile, both types provide the same functionality.

These types of WMRs are capable of changing the direction of their base linear velocity while the base is stationary. This can be instantly performed in the case of holonomic types or in the case of nonholonomic types by changing the direction of the steering wheels over time. This fact allows us to directly control $\psi_{v}$ and have $S^{*}$ as the error states instead of S. Consequently, for this type of WMR, the heading and linear movements are independent. Hence, the controller’s inputs are both $P_{d}$ and $\theta_{d}$, and the body frame B is chosen arbitrarily. For the base path-following (Definition 2) of such WMRs, $s^{\prime }$, and $\kappa_{b}$ of Proposition 1 are $s^{\prime }$, and $\theta_{b}^{\prime }$ given by Equations (30a) and (30b) in Section 4.1, respectively. There are two viable options for $\kappa_{v}$. If the target states is set to be full states $\left(s,S^{*}\right)$, then, as mentioned earlier, $\kappa_{v}$ should be set as $\psi_{d}^{\prime }$ given by Equation (30d). The second option is to set (s,S) as the target states and, therefore, $\kappa_{v}$ should be evaluated using $\psi_{v}^{\prime }$ given by Equation (31a). Table 3 summarize these results.

#### 4.2.2. WMRs with $\delta_{M}=2$

These types of WMRs have limited mobility in their working plane, and the heading and linear movements are dependent. They are either *differential* with δ=(2,0) or *carlike* with δ=(1,1). Both categories have a set of coaxial fixed wheels. The only difference between these categories occurs at the beginning of the path-following. The *differential* type starts the path-following instantly, but the *carlike* type has to steer its steerable wheel according to the start of the path. Aside from this difference, both types provide the same functionality on a smooth path.

**Figure 4:**
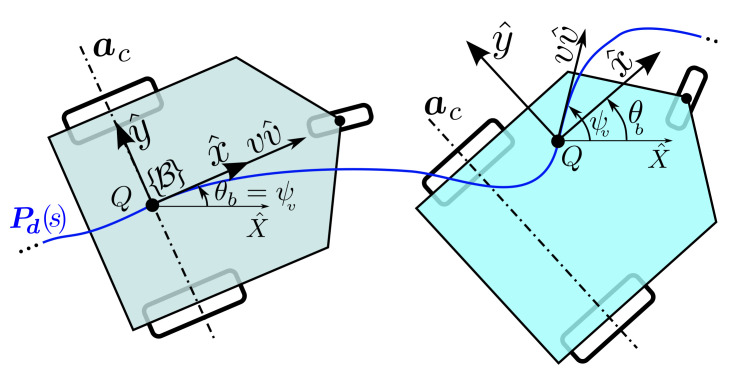
Two WMRs with $\delta_{M}=2$. For the one on the left, the origin of B is on the $a_{c}$ and for the one on the right, the origin is outside of $a_{c}$.

For such WMRs, there is no independent heading control and the progress of the base heading, as shown in Figure 4, is linked to the position of body origin Q. Therefore, in what follows, we strive to derive the control laws for $\omega_{b}$, in the form of $\kappa_{b}v$ of Equation (7b), which observes the kinematic constraints. For a kinematically feasible WMR, all the fixed wheels are coaxial and, therefore, all ${\hat{u}}_{i}$ are on the same line, which we call this common axis $a_{c}$. To derive the constraint equations, set ${\ddot{\phi }}_{i}={\dot{\phi }}_{i}=0$ in Equation (16), and its time derivative, which can be rearranged into

(36a) $$\begin{array}{cc} \omega_{b}\left(d_{i}-{}_{\;}^{B}{\hat{u}}_{i}.\left(\hat{z}\times {}^{B}\ell_{i}\right)\right)= & v\;\;{}_{\;}^{B}{\hat{u}}_{i}.{}_{\;}^{B}\hat{v} \end{array}$$

(36b) $$\begin{array}{cc} v\left(\omega_{b}-\omega_{v}\right)\left(v+l_{i}\omega_{b}\sin\eta_{i}\right)= & \left(d_{i}v_{i}+vl_{i}\cos\eta_{i}\right){\dot{\omega }}_{b}\\ - & \left(d_{i}{\dot{v}}_{i}+\dot{v}l_{i}\cos\eta_{i}\right)\omega_{b}. \end{array}$$

From the above equations, it follows that if Q, the origin of the body frame, is not on the common axis $a_{c}$, a kinematically consistent expression for $\omega_{b}$ may be derived based on Equation (36a). However, when Q is placed on $a_{c}$, Equation (36a) degenerates, since both ${}_{\;}^{B}{\hat{u}}_{i}.{}_{\;}^{B}\hat{v}$ and $d_{i}-{}_{\;}^{B}{\hat{u}}_{i}.\left(\hat{z}\times {}^{B}\ell_{i}\right)$ become zero and Equation (36b) should be employed. If Q has been placed on the common axis, then the right-hand side of Equation (36b) is zero and, in order for the constraint to be valid, $\omega_{b}$ should be set equal to $\omega_{v}$ at all times. In both cases, $\omega_{b}$ is in the form of $\kappa_{b}\left(s,S\right)v$, which are

(37) $$\kappa_{b}=\left\{\begin{array}{cc} {\left(d_{i}-{}_{\;}^{B}{\hat{u}}_{i}.\left(\hat{z}\times {}^{B}\ell_{i}\right)\right)}^{-1}{}_{\;}^{B}{\hat{u}}_{i}.{}_{\;}^{B}\hat{v} & Q\;\mathrm{is}\;\mathrm{not}\;\mathrm{on}\;a_{c}\\ \kappa_{v} & Q\;\mathrm{is}\;\mathrm{on}\;a_{c}. \end{array}\right.$$

Finally, based on Equation (36b), if Q is on the common axis of the fixed wheels $a_{c}$, then the heading is tangent to (or has a constant misalignment with) the footprint of Q, which, in the case of path-following, is eventually $\psi_{t}$. In this case, the WMR is not able to instantly provide any arbitrary $\psi_{v}$; hence, only the full states path-following (s,S) are possible. Consequently, for the base path-following, $s^{\prime }$ is evaluated using Equation (30a) and $\kappa_{b}$, and $\kappa_{v}$ are both set to be $\psi_{v}^{\prime }$ given by Equation (31a). On the other hand, if Q is not placed on $a_{c}$ then, based on Equation (37), $\omega_{b}$ can be used to achieve any arbitrary $\psi_{v}$. Consequently, both the full states’ path-following (s,S), and the reduced states’ path-following $\left(s,S^{*}\right)$ are possible. For the base path-following, $s^{\prime }$ and $\kappa_{b}$ are evaluated using Equations (30a) and (37), respectively. $\kappa_{v}$ is set to $\psi_{v}^{\prime }$ given by Equation (31a) in the full states’ case or $\psi_{d}^{\prime }$ given by Equation (30d) in the case of reduced states. These results are also listed in order in Table 3.

### 4.3. Analysis of Steering Wheels Singularities

In Section 4.1, we provided a stable path-following controller for the base path-following system defined in Definition 2. Next, we incorporated the constraints of Section 3.2 to map the base control signals to the wheels’ velocities. The path-following of a WMR with a stable base controller is stable if there is no singularity in the mapping from the base onto the wheel. Hence, in this section, we elaborate on the singularities of the mapping and how the bounded velocity path-following algorithm treats and resolves those singularities, which specifically occurs with centered steering wheels.

As mentioned in the introduction, one way of examining the singularity of the steering wheels is by studying the base ICR. As the ICR moves closer to a wheel axis, the driving velocity of that wheel decreases and its steering velocity unboundedly increases. When the ICR coincides with the wheel’s steering axis, the steering angle becomes undefined and singular. Here, we study this situation both geometrically and analytically based on the WMR’s path-following.

**Figure 5:**
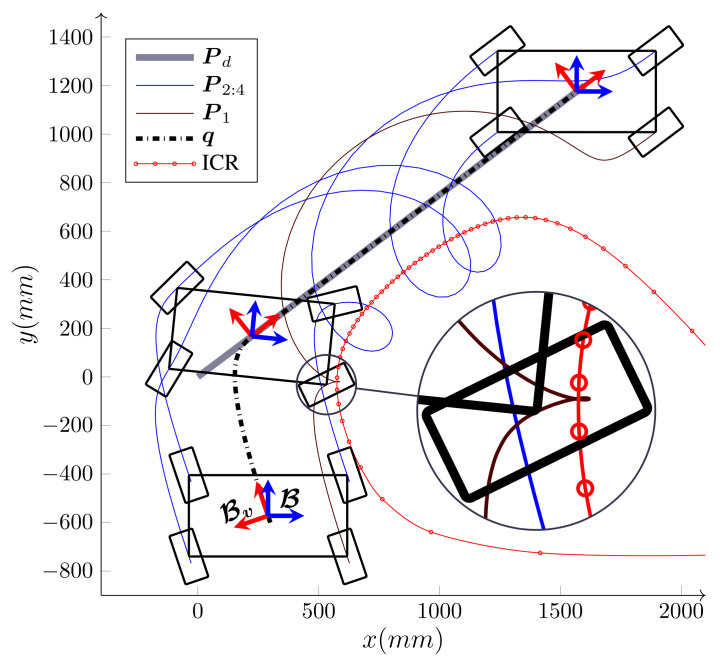
A nonholonomic omnidirectional WMR with four steering wheels following the desired path P(d), which is a straight line, while turning around itself.

Figure 5 shows the path-following of a WMR with four steering wheels that follow a straight line $P_{d}$, during which it rotates around itself for 2π. As shown in the magnified area of the figure, during the operation, as the body ICR moves close to the first wheel, the curvature of $P_{1}$, which is the wheel’s footprint, increases; therefore, for the wheel to *keep up* with the rest of the WMR, it has to increase its steering velocity to pass the tight curvature. At the singularity, the ICR coincides with the steering axis, the curvature of the wheel’s path becomes infinite and the steering direction becomes undefined. Theretofore, geometrically, the kinematic mapping between the base and a wheel becomes singular when a smooth path and movement toward a base result in an infinite curvature for the wheel’s path.

In Section 3.2, for the closed-loop system, we derived the driving and steering velocities, $v_{i}$ and $\phi_{i}$, in the form of $v_{i}^{\prime }v$ and $\phi_{i}^{\prime }v$, respectively. The curvature of $P_{i}$, the *i*th steering wheel’s path, denoted as $\kappa_{i}$, becomes $\kappa_{i}=MM\phi_{i}^{\prime }\;/\;v_{i}^{\prime }$ and can be written as

(38) $$\kappa_{i}=\frac{\kappa_{b}^{\prime }l_{i}\cos\eta_{i}+\left(\kappa_{v}-\kappa_{b}\right)\left(1+l_{i}k_{b}\sin\eta_{i}\right)}{{\left(1+\kappa_{b}^{2}l_{i}^{2}+2l_{i}\kappa_{b}\sin\eta_{i}\right)}^{\frac{3}{2}}}.$$

The singularity occurs when the denominator of the above equation becomes zero; that is, when $\sin\eta_{i}=-\mathrm{sgn}\kappa_{b}$ and $|\kappa_{b}|=MM1\;/\;l_{i}$.

**Figure 6:**
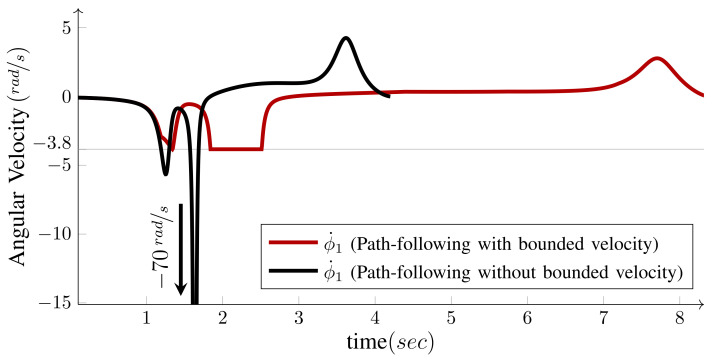
First wheel angular velocity ${\dot{\phi }}_{1}$, with and without bounded velocity.

One of the significant benefits of the bounded velocity path-following presented in Algorithm 1 is that it properly handles the steering wheels’ singularities. As the wheel moves close to its singular configuration $\phi_{i}^{\prime }$ unboundedly increases; therefore, based on Equation (25), that is, $v^{\left(n_{d}+i\right)}={\dot{\phi }}_{i}^{\left(\max\right)}\;/\;|\phi_{i}^{\prime }|$, the velocity candidate $v^{\left(n_{d}+i\right)}$, is reduced to limit ${\dot{\phi }}_{i}$ to its maximum. This, in turn, leads to the reduction in the base speed *v*. Hence, as the WMR moves close to its singular configuration, it reduces its speed and allows the steering wheel to make a tight turn. Figure 6 demonstrates this procedure for the path-following scenario depicted in Figure 5. If the WMR falls right into the singular configuration, $\phi_{i}^{\prime }$ becomes infinity and the robot stops. At this configuration, the curvature of $P_{i}$ is infinity and the path has inward and outward tangents at the singular point. This configuration can be seen as the start of a new path-following; the WMR reorientates its singular wheel from the inward tangent to the outward tangent and starts a new path-following.

## 5. Experimental and Simulation Results

**Figure 7:**
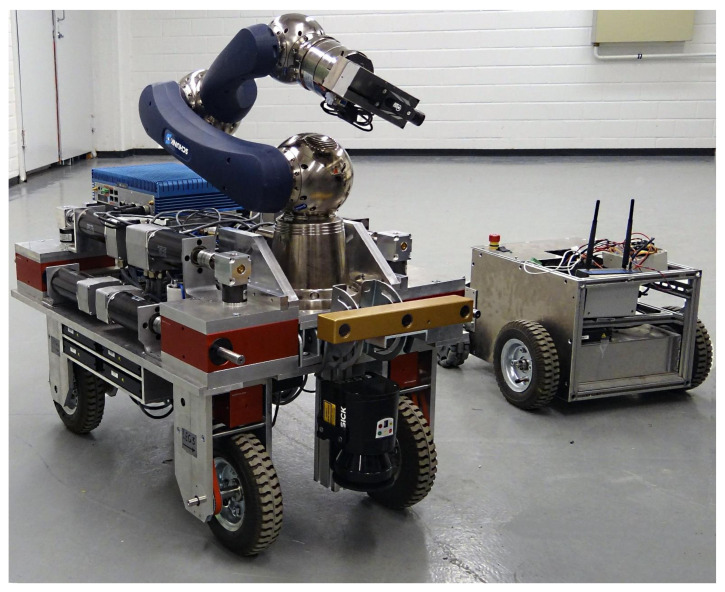
*Experimental setups*: iMoro (**left**): a four-wheel independently steering WMR (δ=(1,2)), and LabRat (**right**): a differential drive WMR (δ=(2,0)) with active fixed wheels at the rear.

In this section, we demonstrate some simulation and experimental data of the presented path-following controller in action. The results are for four categoiesy of WMR. The experimental setup consists of two WMRs, shown in Figure 7. The robot in the left is a four-wheel, independently steered mobile manipulator called iMoro that is a nonholonomic omnidirectional WMR (δ=(1,2)), also known as a two-steer. By manually fixing the steering of the rear wheels (setting ${\dot{\phi }}_{i}=0$), it can also emulate the car-like type (δ=(1,1)). In this case, the steering of the front wheels is naturally governed by the path-following based on the Ackerman principal. This feature was incorporated to test the algorithm for the car-like WMRs. For the last type, the WMR to the right of Figure 7, called LabRat, was employed, which is a differential drive mobile robot (δ=(2,0)) and represents the unicycle kinematics. The path-following controller, namely, the Algorithm 1, was implemented on these WMRs in a real-time Linux environment based on [44]. This section is divided into three case studies; each focuses on different types of WMR and different scenarios (Videos of some of the experiments are provided in the multimedia attachment). Finally, we finish this section by a brief discussion on some of the restrictions of the presented framework.

### 5.1. Case Study I: δ=(3,0) and δ=(2,0)

**Figure 8:**
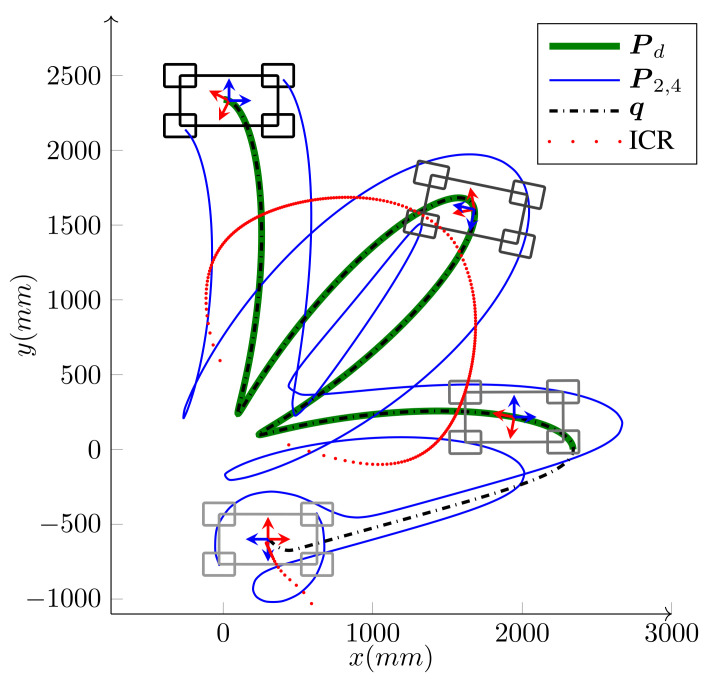
*Simulation*: Path-following with large initial errors of a WMR with four Swedish wheels (δ=(3,0)). It seeks and follows the path $P_{d}$, while correcting its heading from the initial error of $-180^{\circ }$ to the desired heading of $360^{\circ }$ at the end of the path.

**Figure 9:**
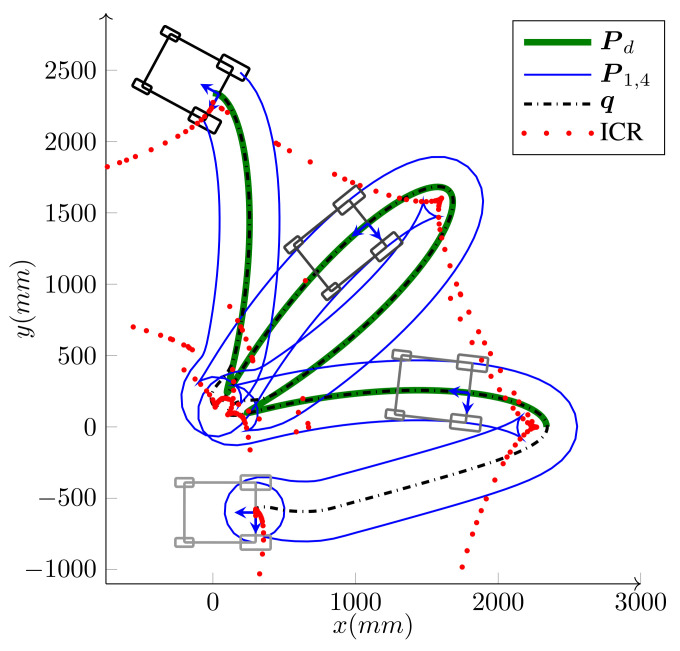
*Experiment*: Bounded velocity path-following with large initial errors for LabRat WMR (δ=(2,0)). It seeks and follows the path $P_{d}$ while correcting its heading from the initial heading error of $-180^{\circ }$ toward the path tangent angle $\psi_{t}\left(s\right)$.

The simulation was performed on a holonomic omnidirectional WMR (δ=(3,0)). The WMR has the same architecture as iMoro, but the steering wheels are virtually replaced by Swedish wheels of the same radii. Figure 8 and Figure 9 portray the path-following scenario, the base footprint q, and the two of the wheels’ paths for the holonomic type (simulation) and the unicycle type (experiment using Labrat), respectively. As shown in the figures, the desired path is smooth, but has sharp turns and, hence, large curvatures at some of its points, which challenges the agility of the controller.

**Figure 10:**
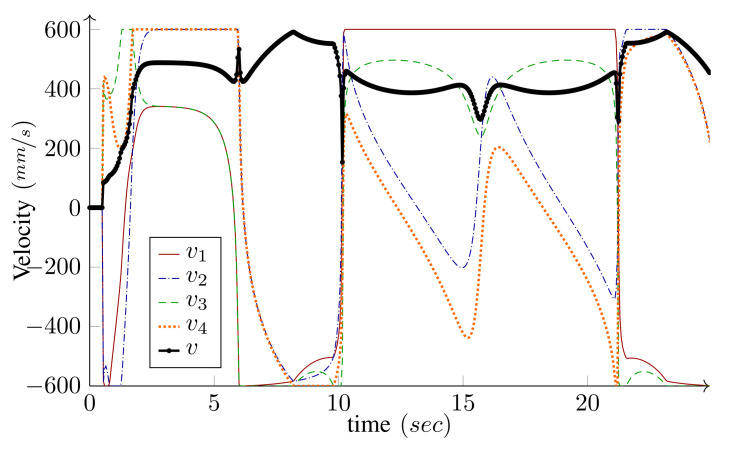
*Simulation*: Driving velocities $v_{i}$, and the base speed *v*, for path-following of a WMR with four Swedish wheels depicted in Figure 8.

**Figure 11:**
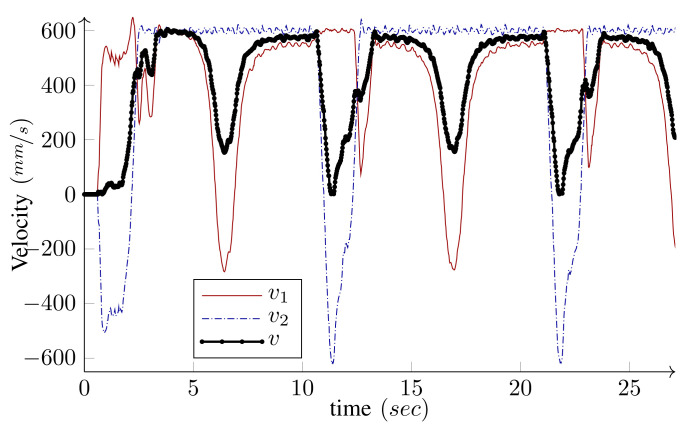
*Experiment*: Driving velocities $v_{1}$ and $v_{2}$, and the base speed *v*, for the bounded velocity path-following of LabRat (δ=(2,0)).

We have intentionally set large initial errors to demonstrate the performance of the controller. The WMR is two meters off the starting point of the path and faces away from it (initial heading error is $-180^{\circ }$). For the holonomic type, independent heading control is possible and the desired heading is $360^{\circ }$ by the end of the path. Conversely, for the unicycle type ($\delta_{M}=2$), independent heading control is not possible. Since the origin of the body frame is on the common axis of the fixed wheels, the heading and the linear velocity angle are the same. Hence, along with the base path-following, the algorithm corrects the initial heading error and the base heading follows the tangent of the path. As shown in the figures, the controller can maneuver the robot toward, and asymptotically onto, the path. The maximum driving velocity was set to 600MMmm/s and the base speed was selected as $v=v^{\left(max\right)}$ given by Equation (26) in Algorithm 1. Figure 10 and Figure 11 present the wheels’ driving velocities $v_{i}$ and the base speed *v*, for the holonomic, and unicycle type, respectively. As shown in the figures, the bounded velocity algorithm duly scales the base speed so that none of the wheels exceed their maximum driving velocity.

As shown in Figure 11, LabRat makes sharp turns by setting the velocities of both wheels to their maximum values but with different signs, which implies that one is driving forward and another is running backward. Hence, the base speed becomes almost zero and the velocity difference leads to the angular velocity that is needed for the turn. Collectively, these experiments demonstrate the agility of the proposed path-following algorithm to steadily realize sharp maneuvers without relying on switching procedures.

### 5.2. Case Study II: δ=(1,2)

**Figure 12:**
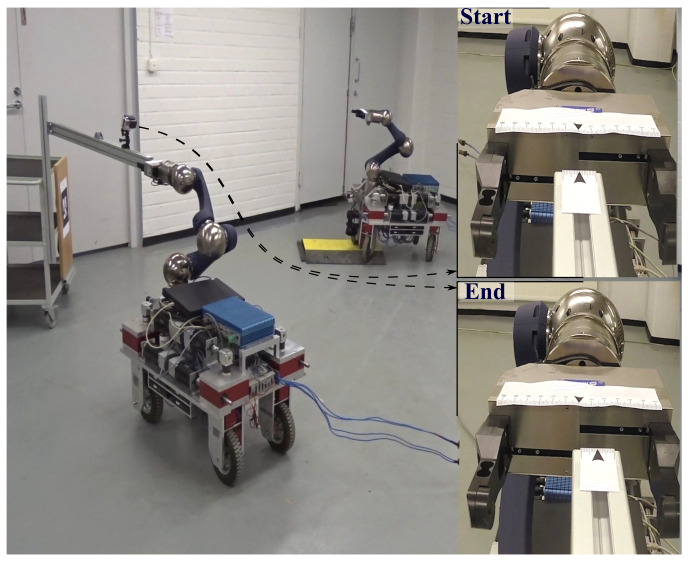
*Experiment*: The repeatability of the path-following controller. The robot starts from the grasping position marked by ”Start“ follows the path shown in Figure 13 and returns close to the initial pose.

**Figure 13:**
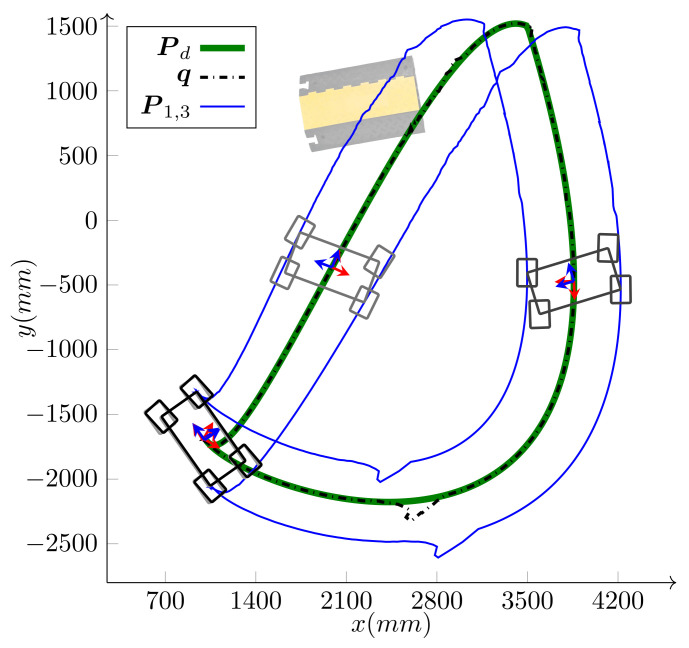
*Experiment*: The desired path and the localization feedback of the WMR, performing the task shown in Figure 12 (The ramp image is shown for the purpose of clarity and does not represent the exact position of the ramp).

This case study focuses on the emulation of a manipulation task with iMoro in the two-steer mode. As shown in Figure 12, the manipulation task consists of grasping the tip of a T-Slot aluminum profile, which is mounted on and extended from a wheeled table. A marker attached on the table is detected by a camera mounted on-board at the front of iMoro. The inertial frame is set on the marker and the pose of the tip is known with respect to the frame, while the wheeled table is placed randomly in the room. At the start, the WMRs’ fingers are about to grasp the tip. It is desired for the WMR to start from this configuration, follow a desired tear shaped path around the room (Figure 13) and creturn to the exact same initial grasping configuration, which provides a means of investigating the repeatability of the system. In this experiment, the localization module consists of sensor fusion between vision data and wheel odometry (more information is given in [45]).

**Figure 14:**
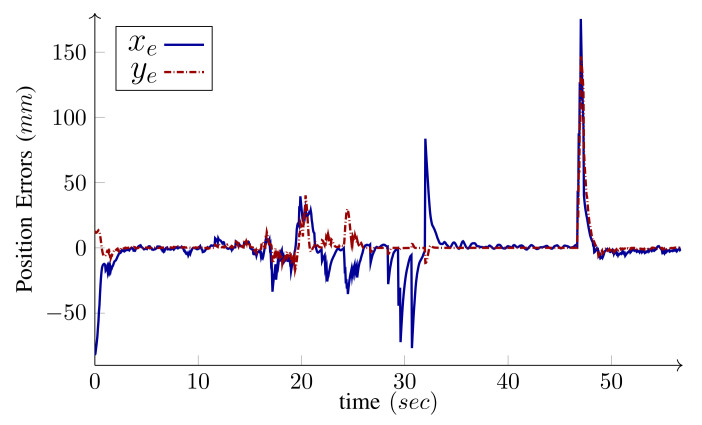
*Experiment*: Position errors in *x* and *y* directions of the inertial frame for the scenario depicted in Figure 13. It shows the disturbances due to the robot moving on a ramp and the localization jump due accumulated error of wheels’ dead reckoning.

To investigate the effects of uncertainties and disturbances, a cable protector ramp is placed somewhere on the path. The WMR driving up and down the ramp precipitates temporary but heavy localization disturbances that are evident in Figure 14 between 20 s and 30 s. Moreover, the independent desired heading is designed such that, for some time, the marker is out of the camera’s field of view and the localization relies solely on wheel odometry. Once the marker returns into the camera’s view, a sudden jump appears in the localization due to the accumulated drifting of the wheels’ dead reckoning, which is also apparent in the figure before 50 s. Since this happens close to the end of the path, the controller has little time to correct the absolute error and bring the robot back to the initial configuration.

The two images on the right side of Figure 12 are from a separate camera mounted on the aluminum profile that show the position of the gripper’s fingers at the start and end of the path-following, which attests to the successful return of the WMR to the initial configuration with around 15 mm of final error. This is also evident in Figure 13 and Figure 14, which show the appearance of the errors and disturbances, and the controller’s pertinent compensation. This experiment was repeated nine times with the ramp placed on several different locations on the path. The WMR successfully returned to the initial grasping configuration with the maximum error of 25 mm. Therefore, while the localization relying on vision and wheel odometry is very noisy, the control system shows sufficient robustness against this noise.

**Figure 15:**
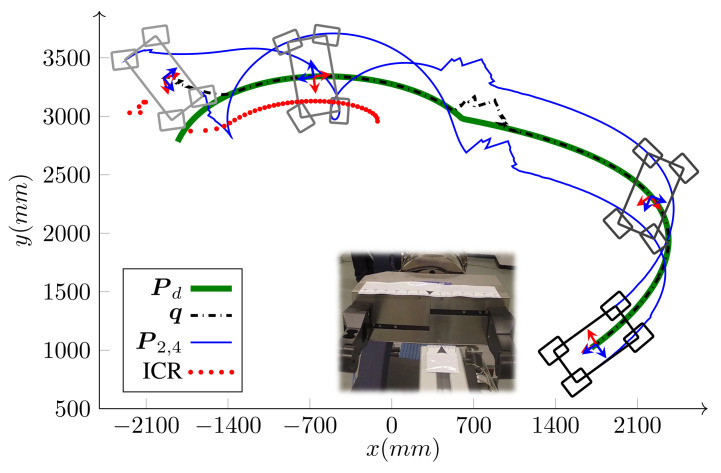
*Experiment*: Bounded velocity path-following with independent heading control of iMoro WMR (δ=(1,2)). It seeks and follows the path $P_{d}$ while correcting its heading from its initial heading to the desired heading of $360^{\circ }$ at the end of the path.

Another scenario using the same setup was also implemented to assess the controller’s practical ability to bound the velocities and alleviate the singularities. In this scenario, the robot has to follow a given path, as shown in Figure 15 that ends in the same grasping configuration as before. However, during the path-following, the WMR is required to make a turn around itself, which not only pushes the platform near its singular configuration but also moves the marker out of the camera’s field of view for some time. As illustrated in Figure 15, the turn happens somewhere near the start of the path, with the base ICR moving closer to one of the wheels steering axis. During the turn, the vision is lost and the accumulated error during this phase results in a localization jump once the camera can see the marker again. However, as shown in the figure, the controller manages to compensate for the errors and usher the robot toward the final grasping pose.

**Figure 16:**
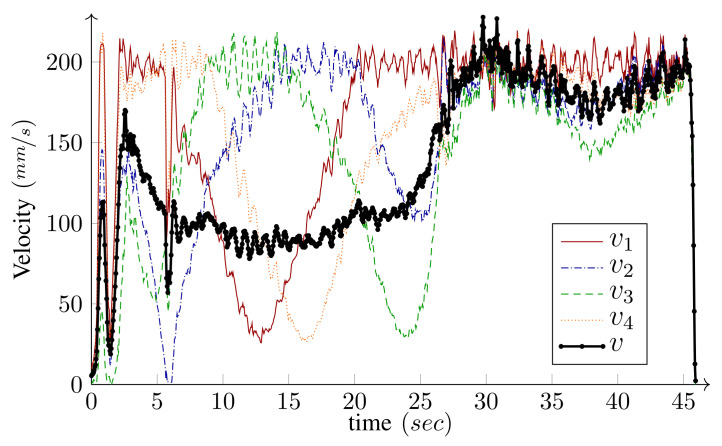
*Experiment*: Driving velocities $v_{i}$, and the base speed *v*, for the bounded-velocity path-following of iMoro depicted in Figure 15. The maximum driving velocity for all the wheels ($v_{i}^{\left(\max\right)}$) are set as 200 mm/s. The base speed is selected to be $v=v^{\left(\max\right)}$, given by Equation (26) in Algorithm 1.

**Figure 17:**
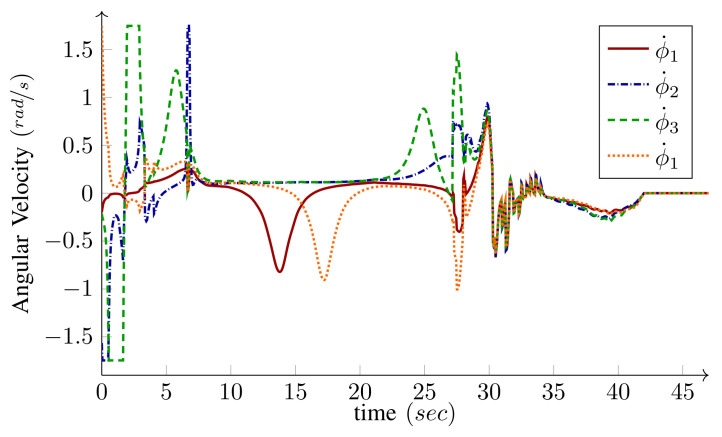
*Experiment*: Steering velocities ${\dot{\phi }}_{i}$ for the bounded velocity path-following of iMoro depicted in Figure 15. The maximum steering velocity for all the wheels (${\dot{\phi }}_{i}^{\left(\max\right)}$) are set as 1.9 rad/s (110 deg/s).

The base speed is selected to be $v=v^{\left(max\right)}$ given by Equation (26) in Algorithm 1; therefore, at least one of the wheels runs with its maximum driving or steering velocity. Comparing Figure 16 and Figure 17, it is clear that, most of the time, at least one of the wheels drives with its maximum driving velocity. However, when a tight turn is needed, the maximum velocity steadily changes from driving to steering, as shown in Figure 17. Notice that, near the beginning of the path, iMoro moves close to its singular configuration a couple of times, which corresponds to some of the peaks in the steering velocities. Figure 15 shows the high curvature of one of the wheels’ footprint and the closeness of the body ICR to the wheel’s steering near the singular configuration.

### 5.3. Case Study III: δ=(1,1)

The last case study focuses on the path-following of car-like WMRs (δ=(1,1)) and, specifically, their bounded steering control. In this paper, we assumed that the steering wheels are free-turn. This assumption can easily be alleviated for some types of WMRs. Generally speaking, limited steering for WMRs with a degree of steerability greater than one ($\delta_{s}=2$) is not favorable. This limitation greatly decreases the maneuverability of the platform to the point that it questions the benefits of allocating extra resources to obtain a WMR with $\delta_{s}>1$. For example, the independence of heading and linear motion is greatly compromised and most of the results in the previous case study would not be possible. However, for configurations with $\delta_{s}=1$, such as car-like WMRs, a limited steering range for steering wheels is common and widely used. Therefore, in this section we present a straightforward approach to account for bounded steering in car-like mode.

**Figure 18:**
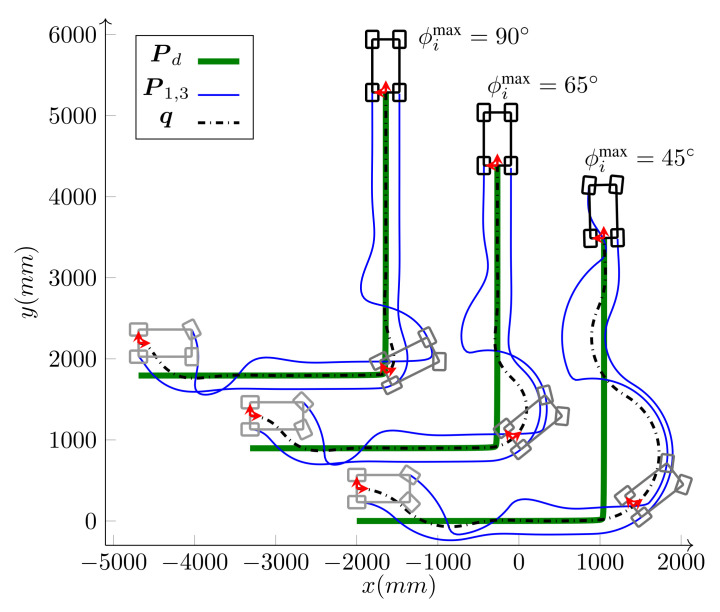
*Experiment*: Path-following of iMoro in car-like mode with three steering limits: $\phi_{i}^{\max}=\left\{45^{\circ },65^{\circ },90^{\circ }\right\}$.

This experiment has been performed on iMoro. While the steering wheels on iMoro are free-turn, virtual limits are set to emulate bounded steering. Figure 18 shows the path-following of iMoro in car-like mode with three steering limits: $\phi_{i}^{\max}=\left\{45^{\circ },65^{\circ },90^{\circ }\right\}$. The desired path is smooth but has a very high curvature at its turning point. The derivation of virtual bounds on control signals to achieve bounded steering is as follows. As shown in the figure, the body frame is on the common axis of the fixed wheels. Therefore, based on Equation (37) and the kinematic constraints of Section 3.2, the velocity constraint $v_{i}{}_{\;}^{B}{\hat{v}}_{i}=v{}_{\;}^{B}\hat{v}+\omega_{b}\left(\hat{z}\times {{}_{\;}^{B}\ell }_{i}\right)$ can be simplified to

(39) $${}_{\;}^{B}{\hat{v}}_{i}\left(\phi_{i}\right)=\frac{{}_{\;}^{B}\hat{v}+\kappa_{v}\left(\hat{z}\times {{}_{\;}^{B}\ell }_{i}\right)}{\left|\right|{}_{\;}^{B}\hat{v}+\kappa_{v}\left(\hat{z}\times {{}_{\;}^{B}\ell }_{i}\right){\left|\right|}_{2}}.$$

In the above equation, ${}_{\;}^{B}\hat{v}$ is known and constant (in case of Figure 18 it is ${\left[1\;0\;0\right]}^{T}$). Replacing $\phi_{i}$ with the front wheels’ steering limit $\phi_{i}^{\max}$, the above equation can be solved for the maximum $\kappa_{v}$, namely $\kappa_{v}^{\max}>0$. Therefore, the bounded control signal ${\bar{\kappa }}_{v}$ that is used to derive actuator commands can be be found by saturating the output of the controller for $\kappa_{v}$, using

(40) $${\bar{\kappa }}_{v}=\left\{\begin{array}{cc} \kappa_{v}\; & |\kappa_{v}|\leq \kappa_{v}^{\max}\\ \mathrm{sgn}\left(\kappa_{v}\right)\kappa_{v}^{\max}\; & |\kappa_{v}|>\kappa_{v}^{\max} \end{array}\right.,$$

in which sgn(x) is the sign function. Note that, for the case where the base frame is not on the common axis of the fixed wheels, based on the first case of Equation (37), a similar procedure can be followed to derive the corresponding bound for the desired velocity direction ${}_{\;}^{B}\hat{v}\left(\psi_{d}\right)$. In this case, the virtual steering bounds are achieved by using the reduced-state model $S^{*}$ and the saturated value of $\psi_{d}$.

### 5.4. Restrictions

As presented in the results of this section, the proposed universal method navigates the WMRs while keeping the driving and steering actuators within their velocity boundaries. This approach is generally suitable in conjunction with a path-planner that generates obstacle-free paths for the WMR. While this approach is capable of navigating the robot toward the desired path, even when the errors are very large, this feature has to be used with care with respect to the obstacles that might be present on the corrective path. Moreover, in order to achieve higher velocity limits while performing tight maneuvers, bounding the velocities is not enough; the accelerations should be bounded too. We have presented a bounded acceleration solution for two-steer WMRs, such as iMoro, in [46], and are currently extending the results to cover all types of WMRs.

## 6. Conclusions

In this paper, we presented a universal bounded-velocity path-following algorithm for Wheeled Mobile Robots (WMRs) operating under the condition of pure rolling without skidding. The solution can be applied to various types of WMRs such as car-like, differential drive, and omnidirectional. The versatility of the framework is due to the generic representation of kinematic constraints. This representation accentuates the possibility of having universal controllers for kinematically different WMRs. We employed these results to derive a closed-form time-scaling solution for the base speed that keeps the velocities of the actuators within a set of pre-specified limits. Extending this establishment, we are currently working to enhance our solution to cover bounded accelerations, dynamic uncertainties and employ barrier functions for obstacle avoidance. 

## Figures and Tables

**Table 1:** Parameters and Variables.

Coordinate Frames and Their Basis		
$U\{\hat{X},\hat{Y}\}$		The inertial frame with its origin O
$B\{\hat{x},\hat{y}\}$		The body frame attached to the WMR’s base Q
$B_{v}\left\{\hat{v},\hat{u}\right\}$		The base velocity coordinate frame at Q
$T\{\hat{t},\hat{n}\}$		The Frenet–Serret frame of the desired path, $P_{d}$, at P
$B_{d}\left\{{\hat{x}}_{d},{\hat{y}}_{d}\right\}$		The desired heading coordinate frame at P
Paths’ Parameters and Variables		
*s*		Natural parameter(arc length) of the desired path, $P_{d}$
$\kappa_{d}\left(s\right)$		The curvature of the desired path, $P_{d}$ ($|\kappa_{d}|<\kappa \neq \infty$)
λ		Natural parameter(arc length) of the asymptotic path, $P_{a}$
Position Vectors		
p	≜	${\left[\begin{array}{ccc} p_{x} & p_{y} & 0 \end{array}\right]}^{T}$; the position vector of the virtual target point, P, with respect to O
q	≜	${\left[\begin{array}{ccc} q_{x} & q_{y} & 0 \end{array}\right]}^{T}$; the position vector of the base, Q, with respect to O
$\ell_{i}$		The position of *i*th wheel attachment point, $L_{i}$, to the base with respect to Q
Angles		
$\theta_{b}$		The WMR’s heading angle that defines the body frame B
$\theta_{d}$		The desired heading angle; defines the frame $B_{d}$ at P
$\psi_{v}$		The angle of the base linear velocity direction, $\hat{v}$
$\psi_{d}$		The desired angle for the base linear velocity direction, $\hat{v}$
$\psi_{vb}$	≜	$\psi_{v}-\theta_{b}$
$\psi_{t}$		The tangent angle; defines the desired path tangent vector $\hat{t}$
$\phi_{i}$		The *i*th wheel steering angle
$\eta_{i}$		The angle between the base linear velocity $v\hat{v}$ and the *i*th wheel position vector, $\ell_{i}$
Others		
$\hat{v}$		The direction vector of the base linear velocity at Q
*v*		The speed of the base at Q
${\hat{v}}_{i}$		The direction vector of the *i*th wheel linear velocity
$v_{i}$		The driving speed of the *i*th wheel
$\omega_{b}$	≜	${\dot{\theta }}_{b}$; the base angular velocity
$\omega_{v}$	≜	${\dot{\psi }}_{v}$; the angular rate of $\hat{v}$
$\omega_{vb}$	≜	$\omega_{v}-\omega_{b}$

**Table 2:** GW Parameters for each Type of Wheel.

	Type	Fixed Wheel	Centered Steering Wheel	Caster Wheel	Swedish Wheel
Variable					
${}_{\;}^{B}{\hat{v}}_{i}\left(\phi_{i}\right)$		Fixed	Measurement or Equation (24)	Measurement	Fixed
$d_{i}$		$d_{i}\in R$	$d_{i}=0$	$d_{i}\in R_{\neq 0}$	$d_{i}\in R$
$c_{i}$		$c_{i}\in R$	$c_{i}\in R$	$c_{i}\in R$	$c_{i}\in R$
γ		$\gamma =\frac{\pi }{2}$	$\gamma =\frac{\pi }{2}$	$\gamma =\frac{\pi }{2}$	$\gamma \neq \frac{\pi }{2}$
$r_{sr}$		$r_{sr}=0$	$r_{sr}=0$	$r_{sr}=0$	$r_{sr}\neq 0$

**Table 3:** Customization of the Path-Following.

	Param.	Error States	Desired Inputs	Selection of *Q*	$\kappa_{b}$	$\kappa_{v}$	$s^{\prime }$
$\delta_{M}$							
$\delta_{M}=3$		$S^{*}$	$P_{d}\left(s\right)$, $\theta_{d}\left(s\right)$	Arbitrary	$\theta_{b}^{\prime }$ (30b)	$\psi_{d}^{\prime }$ (30d)	$s^{\prime }$ (30a)
$\delta_{M}=3$		S	$P_{d}\left(s\right)$, $\theta_{d}\left(s\right)$	Arbitrary	$\theta_{b}^{\prime }$ (30b)	$\psi_{v}^{\prime }$ (31a)	$s^{\prime }$ (30a)
$\delta_{M}=2$		$S^{*}$	$P_{d}\left(s\right)$	Not on $a_{c}$ ${}^{1}$	$\psi_{d}^{\prime }$ (30d)	$\psi_{d}^{\prime }$ (30d)	$s^{\prime }$ (30a)
$\delta_{M}=2$		S	$P_{d}\left(s\right)$	Not on $a_{c}$	$\kappa_{b}$ (37)	$\psi_{v}^{\prime }$ (31a)	$s^{\prime }$ (30a)
$\delta_{M}=2$		S	$P_{d}\left(s\right)$	On $a_{c}$	$\psi_{v}^{\prime }$ (31a)	$\psi_{v}^{\prime }$ (31a)	$s^{\prime }$ (30a)

${}^{1}$$a_{c}$ is the common axis of the fixed wheels.

## Data Availability

Not applicable.

## Supplementary Materials

The following are available online at https://www.mdpi.com/article/10.3390/s21227642/s1.

## References

[1] Morin P., Samson C. (2008). Motion control of wheeled mobile robots. Springer Handbook of Robotics.

[2] Park K., Chung H., Lee J.G. (2000). Point stabilization of mobile robots via state-space exact feedback linearization. Robot. Comput.-Integr. Manuf..

[3] Hwang C.L., Wu H.M. (2013). Trajectory tracking of a mobile robot with frictions and uncertainties using hierarchical sliding-mode under-actuated control. IET Control Theory Appl..

[4] Fossen T., Pettersen K.Y., Galeazzi R. (2015). Line-of-sight path following for Dubins paths with adaptive sideslip compensation of drift forces. IEEE Trans. Control Syst. Technol..

[5] Bullo F., Lynch K.M. (2001). Kinematic controllability for decoupled trajectory planning in underactuated mechanical systems. IEEE Trans. Robot. Autom..

[6] Van Loock W., Pipeleers G., Diehl M., De Schutter J., Swevers J. (2014). Optimal Path Following for Differentially Flat Robotic Systems Through a Geometric Problem Formulation. IEEE Trans. Robot..

[7] Debrouwere F., Van Loock W., Pipeleers G., Dinh Q.T., Diehl M., De Schutter J., Swevers J. (2013). Time-optimal path following for robots with convex–concave constraints using sequential convex programming. IEEE Trans. Robot..

[8] Akhtar A., Nielsen C., Waslander S.L. (2015). Path following using dynamic transverse feedback linearization for car-like robots. IEEE Trans. Robot..

[9] Ambrosino G., Ariola M., Ciniglio U., Corraro F., De Lellis E., Pironti A. (2009). Path generation and tracking in 3-D for UAVs. IEEE Trans. Control Syst. Technol..

[10] Yamasaki T., Balakrishnan S. (2010). Sliding mode-based pure pursuit guidance for unmanned aerial vehicle rendezvous and chase with a cooperative aircraft. Proc. Inst. Mech. Eng. Part G J. Aerosp. Eng..

[11] Nelson D.R., Barber D.B., McLain T.W., Beard R.W. (2007). Vector field path following for miniature air vehicles. IEEE Trans. Robot..

[12] Sujit P., Saripalli S., Borges Sousa J. (2014). Unmanned aerial vehicle path following: A survey and analysis of algorithms for fixed-wing unmanned aerial vehicless. IEEE Control Syst..

[13] Samson C. (1993). Time-varying feedback stabilization of car-like wheeled mobile robots. Int. J. Robot. Res..

[14] Aicardi M., Casalino G., Bicchi A., Balestrino A. (1995). Closed loop steering of unicycle like vehicles via Lyapunov techniques. IEEE Robot. Autom. Mag..

[15] Micaelli A., Samson C. (1993). Trajectory Tracking for Unicycle-Type and Two-Steering-Wheels Mobile Robots.

[16] Soetanto D., Lapierre L., Pascoal A. Adaptive, non-singular path-following control of dynamic wheeled robots. Proceedings of the 42nd IEEE Conference on Decision and Control.

[17] Lapierre L., Soetanto D., Pascoal A. (2006). Nonsingular path following control of a unicycle in the presence of parametric modelling uncertainties. Int. J. Robust Nonlinear Control.

[18] Lapierre L., Indiverri G. Path-Following Control of a Wheeled Robot under actuation saturation constraints. Proceedings of the IAV07 Conference.

[19] Lapierre L., Zapata R., Lepinay P. (2007). Combined path-following and obstacle avoidance control of a wheeled robot. Int. J. Robot. Res..

[20] Kaminer I., Pascoal A., Xargay E., Hovakimyan N., Cao C., Dobrokhodov V. (2010). Path following for small unmanned aerial vehicles using L1 adaptive augmentation of commercial autopilots. J. Guid. Control. Dyn..

[21] Encarnaçao P., Pascoal A. 3D path following for autonomous underwater vehicle. Proceedings of the 39 th IEEE Conference on Decision and Control.

[22] Ghabcheloo R., Hyvonen M. Modeling and motion control of an articulated-frame-steering hydraulic mobile machine. Proceedings of the 17th Mediterranean Conference on Control and Automation (MED’09).

[23] Bibuli M., Bruzzone G., Caccia M., Lapierre L. (2009). Path-following algorithms and experiments for an unmanned surface vehicle. J. Field Robot..

[24] Kanjanawanishkul K., Zell A. Path following for an omnidirectional mobile robot based on model predictive control. Proceedings of the IEEE International Conference on Robotics and Automation (ICRA’09).

[25] Thuilot B., d’Aandrea Novel B., Micaelli A. (1996). Modeling and feedback control of mobile robots equipped with several steering wheels. IEEE Trans. Robot. Autom..

[26] Connette C.P., Pott A., Hagele M., Verl A. Control of an pseudo-omnidirectional, non-holonomic, mobile robot based on an ICM representation in spherical coordinates. Proceedings of the 47th IEEE Conference on Decision and Control (CDC 2008).

[27] Song J.B., Byun K.S. (2009). Steering control algorithm for efficient drive of a mobile robot with steerable omni-directional wheels. J. Mech. Sci. Technol..

[28] Moore K.L., Davidson M., Bahl V., Rich S., Jirgal S. Modelling and control of a six-wheeled autonomous robot. Proceedings of the IEEE 2000 American Control Conference.

[29] Connette C., Parlitz C., Hägele M., Verl A. Singularity avoidance for over-actuated, pseudo-omnidirectional, wheeled mobile robots. Proceedings of the IEEE International Conference on Robotics and Automation (ICRA).

[30] Schwesinger U., Pradalier C., Siegwart R. A novel approach for steering wheel synchronization with velocity/acceleration limits and mechanical constraints. Proceedings of the 2012 IEEE/RSJ International Conference on Intelligent Robots and Systems (IROS).

[31] Cousins S. (2010). Ros on the pr2 [ros topics]. IEEE Robot. Autom. Mag..

[32] Graf B., Reiser U., Hagele M., Mauz K., Klein P. Robotic home assistant Care-O-bot® 3-product vision and innovation platform. Proceedings of the 2009 IEEE Workshop on Advanced Robotics and its Social Impacts (ARSO).

[33] Dietrich A., Wimbock T., Albu-Schaffer A., Hirzinger G. (2012). Reactive Whole-Body Control: Dynamic Mobile Manipulation Using a Large Number of Actuated Degrees of Freedom. IEEE Robot. Autom. Mag..

[34] Bak T., Jakobsen H. (2004). Agricultural robotic platform with four wheel steering for weed detection. Biosyst. Eng..

[35] Cariou C., Lenain R., Thuilot B., Berducat M. (2009). Automatic guidance of a four-wheel-steering mobile robot for accurate field operations. J. Field Robot..

[36] Frémy J., Ferland F., Lauria M., Michaud F. (2014). Force-guidance of a compliant omnidirectional non-holonomic platform. Robot. Auton. Syst..

[37] Connette C., Hagele M., Verl A. Singularity-free state-space representation for non-holonomic, omnidirectional undercarriages by means of coordinate switching. Proceedings of the 2012 IEEE/RSJ International Conference on Intelligent Robots and Systems (IROS).

[38] Connette C., Pott A., Hägele M., Verl A. Addressing input saturation and kinematic constraints of overactuated undercarriages by predictive potential fields. Proceedings of the IEEE/RSJ International Conference on Intelligent Robots and Systems (IROS).

[39] Oftadeh R., Aref M.M., Ghabcheloo R., Mattila J. Bounded-velocity motion control of four wheel steered mobile robots. Proceedings of the 2013 IEEE/ASME International Conference on Advanced Intelligent Mechatronics (AIM).

[40] Oftadeh R., Ghabcheloo R., Mattila J. A novel time optimal path following controller with bounded velocities for mobile robots with independently steerable wheels. Proceedings of the 2013 IEEE/RSJ International Conference on Intelligent Robots and Systems (IROS).

[41] Oftadeh R., Ghabcheloo R., Mattila J. A time-optimal bounded velocity path-following controller for generic Wheeled Mobile Robots. Proceedings of the 2015 IEEE International Conference on Robotics and Automation (ICRA).

[42] Campion G., Bastin G., Dandrea-Novel B. (1996). Structural properties and classification of kinematic and dynamic models of wheeled mobile robots. IEEE Trans. Robot. Autom..

[43] Osmolovskii N., Maurer H. (2012). Applications to Regular and Bang-Bang Control.

[44] Oftadeh R., Aref M.M., Ghabcheloo R., Mattila J. Unified framework for rapid prototyping of linux based real-time controllers with matlab and simulink. Proceedings of the 2012 IEEE/ASME International Conference on Advanced Intelligent Mechatronics (AIM).

[45] Aref M.M., Oftadeh R., Ghabcheloo R., Mattila J. Real-time vision-based navigation for nonholonomic mobile robots. Proceedings of the 2016 IEEE International Conference on Automation Science and Engineering (CASE).

[46] Oftadeh R., Ghabcheloo R., Mattila J. Time Optimal Path Following with Bounded Velocities and Accelerations for Mobile Robots with Independently Steerable Wheels. Proceedings of the 2014 IEEE International Conference on Robotics and Automation (ICRA).

